# Differential regulation of myopia progression by ON and OFF stimulation in guinea pigs

**DOI:** 10.1186/s40662-026-00495-z

**Published:** 2026-06-09

**Authors:** Qiqi Xie, Qin Dai, Xiuying Zhu, Xi Chen, Bingyan Shen, Yenan Fang, Xinyu Li, Fan Lu, Xiangtian Zhou, Min Wang

**Affiliations:** 1 State Key Laboratory of Eye Health, Eye Hospital, Wenzhou Medical University, Wenzhou, 325027 China; 2 National Clinical Research Center for Ocular Diseases, Eye Hospital, Wenzhou Medical University, Wenzhou, 325027 China; 3 The Lishui Central Hospital, the Fifth Affiliated Hospital of Wenzhou Medical University, Lishui, 323000 China

**Keywords:** ON and OFF pathways, Guinea pig, Myopia, Choroid, Dopamine

## Abstract

**Background:**

The effects of ON and OFF retinal pathway stimulation on lens-induced myopia (LIM) in mammalian models remain unclear. This study examined refractive, choroidal, and dopamine (DA)-related responses to artificial dynamic ON and OFF stimulation during the development of LIM in guinea pigs.

**Methods:**

Sixty-three guinea pigs were randomly assigned to ON, OFF, and natural control (NC) stimulation for 12 h/day over 7 days. LIM was induced using a − 4.0 D monocular lens. Refraction, axial length (AL), choroidal thickness (ChT), and choroidal blood perfusion (ChBP) were assessed before and after treatment. DA and metabolites were measured by high-performance liquid chromatography (HPLC), interocular differences were calculated, and choroidal proteomes were analyzed using liquid chromatography–tandem mass spectrometry (LC–MS/MS) based bioinformatics analysis.

**Results:**

ON stimulation significantly reduced myopic shift compared with NC and OFF stimulation (Δ refraction: − 2.13 ± 0.40 D vs. − 4.03 ± 0.35 D and − 3.98 ± 0.50 D, respectively; both *P* < 0.01). No significant differences in AL were observed among groups. OFF stimulation uniquely preserved ChBP (ΔChBP: 0.51% ± 2.18%), significantly different from ON (− 6.11% ± 1.76%, *P* = 0.0330) and NC (− 7.78% ± 1.44%, *P* = 0.0057). OFF stimulation also showed less choroidal thinning (ΔChT: − 3.06 ± 2.23 μm) compared to NC (− 12.35 ± 2.7 μm, *P* = 0.0171). In non-lens eyes, ON stimulation produced higher ChBP than NC or OFF stimulation, and ChT changes were positively correlated with ChBP under all conditions (*P* < 0.0001). Retinal 3,4-dihydroxyphenylacetic acid (DOPAC) levels were significantly higher in the ON group (17.65 ± 12.67) than in the NC group (− 25.75 ± 9.23, *P* = 0.0095). Vitreal DA markers showed no significant differences among groups. Proteomic analysis revealed distinct choroidal remodeling signatures among ON, OFF, and NC groups, with ON stimulation associated with the smallest number of differentially expressed proteins and a relatively preserved pattern of collagen and fibrillin expression compared with more pronounced extracellular matrix (ECM) depletion under NC and over-compensatory ECM upregulation under OFF stimulation.

**Conclusions:**

ON and OFF stimulation differentially regulated ocular responses to myopia. ON stimulation was associated with attenuated myopic shift and increased retinal DOPAC levels, suggesting enhanced DA metabolism. OFF stimulation was associated with preserved ChT and ChBP, accompanied by upregulation of ECM components. These findings suggest that effective myopia control strategies may require coordinated engagement of both ON- and OFF-pathway-mediated mechanisms.

**Supplementary Information:**

The online version contains supplementary material available at 10.1186/s40662-026-00495-z.

## Background

Myopia, projected to affect nearly 4.8 billion people by 2050, is a major global public health concern [1]. Consequently, effective myopia control strategies are of substantial public health importance. However, the role of near work remains controversial, with shorter working distances (< 30 cm) and continuous activity (> 30 min) emerging as key risk factors [2]. Beyond optical defocus and accommodation, recent evidence indicates that reading and other visual tasks may influence ocular development by differentially engaging the retinal ON pathway, thereby linking visual behavior to downstream eye growth regulation. In particular, reading-related modulation of retinal pathway activation may have important implications for refractive development and visual health [3].

The retinal ON and OFF pathways exhibit distinct spatial and temporal characteristics: ON pathways respond preferentially to rapid increases in illuminance, whereas OFF pathways respond preferentially to rapid decreases [4, 5]. These pathways can be differentially biased by manipulating the temporal illuminance profiles and contrast polarity, with consequent differential effects on eye growth. Consistent with previous human evidence [6], our earlier work showed that ON and OFF stimuli are approximately balanced in natural viewing, and that reading dark text on a bright background, which preferentially stimulates the OFF pathway, reduced choroidal thickness (ChT) within 1 h, whereas reading bright text on a dark background, which preferentially stimulates the ON pathway, increased ChT [7]. ChT declines with increasing myopia severity and is closely associated with axial length (AL) [8]. Longitudinal data in children with progressive myopia further indicate that ChT may remain relatively stable during early stages of moderate myopic progression but becomes significantly thinner with progression to high myopia [9]. In addition, short-term reading of bright text against a dark background has been associated with transient AL shortening in humans [10]. Since animals cannot read, experimental ON/OFF paradigms have used artificial dynamic ON and OFF stimulation to model contrast-polarity-dependent visual input in chicks [11]. In humans and chickens, short-term ON stimulation increases the choroid, whereas OFF stimulation causes choroidal thinning. However, in chicks, prolonged (7 day) ON/OFF stimulation has been reported to promote myopia development and increase retinal dopamine (DA) production [11], suggesting that the long-term impact of ON/OFF stimulation on eye growth may differ across species and experimental paradigms and remains incompletely understood in mammalian models.

With advances in myopia research, optical coherence tomography (OCT), and OCT angiography (OCTA), the choroid has become a central focus in refractive development studies. Accumulating evidence indicates that the choroid is actively involved in visually guided eye growth and refractive regulation [12]. Zhang et al. [13] demonstrated that ChT and choroidal blood perfusion (ChBP) were significantly reduced in the myopic eyes of guinea pigs, with changes in ChBP preceding choroidal thinning, suggesting that vascular dysregulation may contribute to myopia development. Consistently, clinical and experimental studies have reported reduced ChBP in high myopia[14, 15]. Despite these observations, the specific mechanisms by which the choroid contributes to myopia progression remain unclear, particularly regarding how ON and OFF stimulation modulates ChT and ChBP. Intraocular DA release is widely recognized as a key biomarker in myopia [16]. Prolonged exposure to natural light increases retinal DA secretion and is thought to suppress myopia onset and progression [17]. DA regulates ChBP by activating DA receptors in the choroidal vasculature [18]. These findings raise the possibility that ON/OFF stimulation may influence the development of myopia via the DA–choroid axis, wherein changes in DA release and receptor activation alter choroidal perfusion and structure, ultimately affecting ocular growth.

Previous omics studies in guinea pigs have provided additional evidence that the choroid actively contributes to myopia development. Proteomic analyses have suggested that greater ChT may protect against myopia progression through nitric oxide (NO)-related signaling pathways [19] and that protein expression in choroidal vascular endothelial cells can reflect retinal protein changes during myopia development [20]. Metabolomic analyses further indicate that the retinal pigment epithelium (RPE)/choroid complex may regulate form-deprivation myopia (FDM) through proline consumption involving the pentose phosphate and reductive carboxylation pathways and that intravitreal injection of a DA D2 receptor antagonist can attenuate myopic shift, increase proline concentrations, and activate arginine/proline and purine metabolism pathways [21]. Together, these studies support the choroid as an active regulator of myopia through proteomic and metabolic remodeling, particularly involving NO signaling and proline metabolism. However, it remains unclear how ON and OFF stimulation modulates choroidal proteomic remodeling and DA-related signaling in the context of lens-induced myopia (LIM) context in mammalian models.

Overall, the roles of ON and OFF stimuli in visually guided myopic changes remain poorly defined in guinea pigs. Given that ON/OFF-specific changes in choroidal structure and perfusion, as well as DA release, may contribute to myopic eye growth, we hypothesized that ON and OFF stimulation would differentially modulate refractive and biometric parameters including AL, ChT, ChBP, as well as choroidal proteomic profiles, and DA-related signaling in LIM guinea pigs. Specifically, we aimed to determine whether artificial dynamic ON and OFF stimulation differentially affects LIM progression, induces distinct changes in ChT, ChBP, and choroidal proteomic profiles, and differentially modulates DA and its metabolites in the retina and vitreous. These findings may provide new insights into ON/OFF-pathway–based strategies for myopia prevention and control.

## Methods

### Animal subjects

In this study, 63 pigmented guinea pigs aged 3 weeks, with an average weight of 150–180 g, were purchased from the Laboratory Animal Center of Wenzhou Medical University. Their housing environment was maintained at 25 °C with a 12-h light/12-h dark cycle (light period starting at 8 a.m.). The guinea pigs had ad libitum access to water and a standard chow diet supplemented with fresh vegetables once daily. The Animal Care and Ethics Committee of Wenzhou Medical University (Wenzhou, China) approved the research protocol (Approval ID: wydw2023-0063).

### Biometric measurements

Refraction was assessed without cycloplegia using an eccentric infrared photorefractor [22]. Animals with anisometropia > 2 diopters (D) were excluded from the study. Because ChT and AL undergo diurnal modulation, measurements were always taken between 8 and 11 a.m. In this study, AL was measured using A-scan ultrasonography (A VISO Echograph Class I-Type Bat; Quantel Medical, Clermont-Ferrand, France) after corneal anesthesia with a 0.5% proparacaine hydrochloride solution. To ensure the measurement accuracy and repeatability, the probe was aligned along the visual axis with minimal corneal indentation. The ultrasound frequency and conduction velocity in the eyes were consistent with previously established parameters. The final ocular biometric value for each eye was the average of 10 consecutive measurements, which demonstrated high within-session reproducibility (coefficient of variation, consistently < 1%). OCT and OCTA images were captured using a Spectralis HRA + OCT (Heidelberg Engineering, Heidelberg, Germany). According to the method described by Zhou et al., enhanced depth imaging (EDI) and follow-up mode scans were performed, and images passing through the center of the optic disc with a quality score greater than 30 were included in the final analysis. To control for potential variability due to ocular tilt, all B-scans were standardized to pass vertically through the center of the optic nerve head. Additionally, horizontal tilt was minimized by ensuring that the reflex from the vitreous-retina interface was maximal, and that the RPE layer on either side of the optic disc exhibited equivalent intensity and clarity. A custom program (MATLAB R2017a) was used to determine ChBP and ChT simultaneously [13]. To assess the repeatability of ChT measurements, two experienced operators independently performed measurements on a randomly selected subset of eyes (n = 19 per group, representing approximately 20% of the total sample). Bland–Altman analysis revealed good inter-operator agreement, with bias values ranging from − 2.08 to − 0.98 μm across all conditions and 95% limits of agreement within ± 10 μm. The intraclass correlation coefficient (ICC) was > 0.90 for all measurements, indicating excellent reliability (Supplementary Fig. 3).

### Treatment

#### Myopia induction

Myopia was induced in either the left or right eye randomly assigned using − 4.0 D-negative lenses, with the other eye left untreated to serve as an internal reference. The lenses were affixed to a plastic gasket, which was then affixed to the corresponding hook-side Velcro rings, and attached to the feathers surrounding the eyes of the guinea pigs. The lenses were cleaned twice daily to prevent infection and inflammation while maintaining clear retinal images.

#### Seven days of light stimuli exposure in the LIM animal model

Following lens fixation, the guinea pigs were randomly allocated to three experimental groups (n = 21 per group): natural control (NC), dynamic ON stimulus, and dynamic OFF stimulus. All animals underwent a 7-day regimen of visual stimulation from 8:00 a.m. to 8:00 p.m. daily, followed by complete darkness until the next cycle. Ocular parameters including refraction, AL, ChT, and ChBP were assessed before and after the experimental period. The visual stimulation system was programmed in Visual C + + 8.0, generating approximately 2000 squares exhibiting sawtooth-wave illuminance modulation at 1 Hz [11]. Stimuli were presented using the cage and monitor configuration described in our earlier study [23]: a transparent cage measuring 60 × 60 × 60 cm with four Acer XF270H monitors serving as walls. Each stimulus element was 10 × 10 mm (subtending 57 arcmin; approximately 1.05 cycles/degree at maximum viewing distance), with temporal cycling at ~ 1 Hz. Using a TA632A digital illuminance meter (Suzhou Teans, China), the three stimulation conditions were brightness-matched to 153 ± 10 lx measured at nine cage locations, yielding an average illuminance of ~ 150 lx. For the NC condition, the animals were maintained under standard indoor cage lighting without additional visual stimulation, with the illuminance calibrated to ~ 150 lx.

#### Tissue sample preparation

Each stimulus group was further divided into two subgroups, myopic and control eyes, yielding six subgroups. Since DA levels are dependent on light and circadian cycles, the retina and vitreous were collected 4–6 h after light onset. They were collected under the same illuminance level for each eye and prepared for DA assessments. The proteomic samples comprised choroidal tissues from two guinea pigs, resulting in three samples per subgroup. Freshly enucleated eyes were first placed on ice, and then the anterior segment and lens were removed before the vitreous (n = 15/group), retina (n = 15/group), and choroid (n = 6/group) were gently separated. All tissues were promptly frozen in liquid nitrogen and stored at − 80 ℃ until further analysis.

#### High-performance liquid chromatography (HPLC) detection of DA

After tissue preparation, the catecholamines were quantified using an HPLC-ECD system (Thermo Fisher Scientific, Waltham, MA, USA). Notably, retinal samples were stored at − 80 ℃ before detection. For retinal samples, 20 μL of freshly prepared homogenate (H3ClO4 0.1 M, EDTA Na2 0.1 mM, standard internal DHBA) was added per mg of tissue. Vitreous samples were homogenized at 2 μL/mg wet weight, with 2 × , 3 × , 5 × volumes used when needed (4, 6, or 10 μL/mg), depending on the weight of the tissue. First, the samples were homogenized at − 40℃ using 2 mm zirconia beads at a frequency of 60 Hz for 30 s, repeated four times (Beijing Ltd., Beijing N-9548R). Subsequently, low-temperature centrifugation was performed at 20,000 rpm for 30 min at 4 °C (Thermo Fisher Scientific). The supernatant was collected and stored at − 80 °C before thawing the sample and centrifuging again (20,000 rpm, 30 min, 4 °C). Finally, 10 μL of the resulting supernatant was collected and used for analysis. The target concentration was determined using the internal standard method and the concentration per gram of wet tissue (ng/g wet weight) was calculated.

#### Choroidal protein extraction and digestion

The procedures for protein extraction, digestion, and peptide preparation from choroidal tissues were consistent with the methods described above for retinal samples. In brief, proteins were extracted using SDT lysis buffer, quantified with the BCA assay, and digested via the filter-aided sample preparation (FASP) technique [24]. The resulting peptides were desalted using C18 solid-phase extraction cartridges, concentrated by vacuum centrifugation, and reconstituted in 40 μL of 0.1% formic acid. The peptide concentration was determined by measuring the ultraviolet (UV) absorbance at 280 nm.

#### Protein identification using liquid chromatography–mass spectrometry/mass spectrometry (LC–MS/MS) analysis

LC–MS/MS analysis was performed using a timsTOF Pro mass spectrometer coupled with a NanoElute nanoflow LC system for a 60-min duration, following a previously established methodology [25]. Briefly, the peptide samples were trapped on a reverse-phase column and separated using a C18 reversed-phase analytical column with an acetonitrile gradient. Mass spectrometry detection was conducted in positive ion mode, covering a mass range of m/z 100–1700 and an ion mobility range of 1/k_0_ 0.6–1.6, with 10 cycles of PASEF MS/MS acquisition. Raw MS data from all samples were processed collectively using MaxQuant software (v1.5.3.17), with protein and peptide false discovery rates set at ≤ 0.01. Protein identification was based on razor and unique peptides, and quantification was performed using label-free quantification.

#### Bioinformatics analysis of proteomics data

First, label-free quantification intensity data were filtered, normalized, and missing values were filled using the R package "DEP" to obtain standardized expression data. Differentially expressed proteins (DEPs) were identified by comparing the right and left eyes of the same animal within each group using the test_diff function (*P* < 0.05, |Log_2_ (fold change) |> 1). To maximize the sensitivity of the exploratory pathway analysis, no multiple testing corrections were applied at the initial DEP identification stage. The results were visualized using Venn diagrams and volcano plots. For functional interpretation, Gene Ontology (GO) and Kyoto Encyclopedia of Genes and Genomes (KEGG) enrichment analyses were performed using the clusterProfiler package (Version 4.2.2) with adjusted p-values (*P* < 0.05) to ensure statistical rigor and biological reliability [26]. GO enrichment analysis was conducted across three ontology modules: biological processes (BPs), molecular functions, and cellular components.

### Statistical analysis

Statistical analyses were performed using "GraphPad Prism version 9.4.0" (GraphPad Software, San Diego, CA, USA) and SigmaStat software (San Jose, CA, USA). One-way ANOVA was used to compare the stimulus effects in myopic and control eyes. Pearson’s correlation analysis was used to assess the correlation between changes in refraction and AL in the guinea pigs. Significance was set at *P* < 0.05, and all results were reported as mean ± standard error of the mean (SEM). Bioinformatics analyses were conducted in R using the DEP package (Version 1.20.0) for differential expression and Pearson correlation analyses [27].

## Results

### Differences in biometric parameters between various visual stimulations and induced myopia

**Fig. 1:**
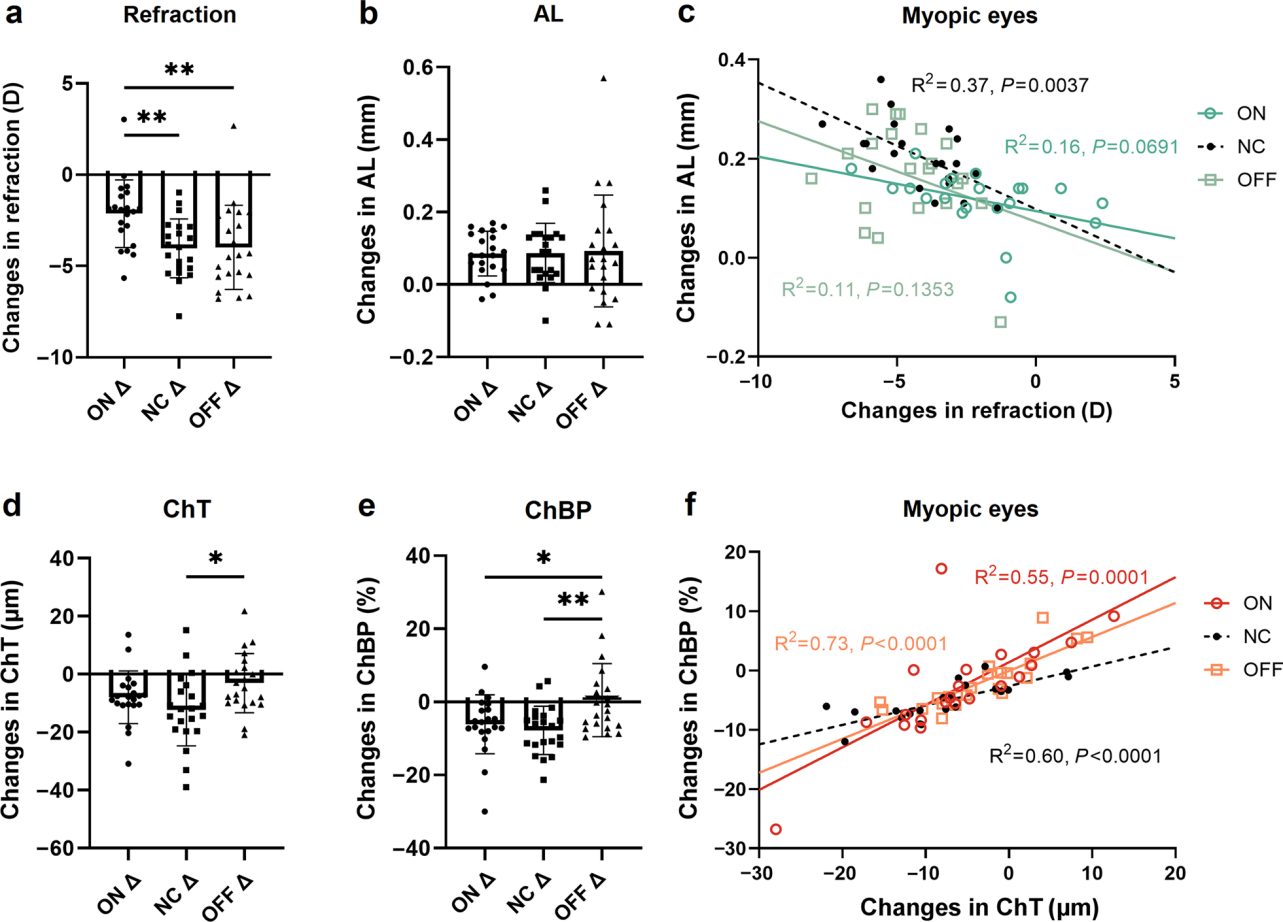
Effects of ON and OFF stimulation on ocular biometric parameters in lens-induced myopia. **a** Changes in refraction expressed as interocular differences (Δ = lens-treated right eye change − untreated left eye change) in the ON, NC, and OFF groups after 7 days of − 4.0 D lens wear. **b** Changes in axial length (AL) expressed as interocular differences in the three groups. No significant differences were detected among groups (all *P* > 0.05). **c** Correlation between changes in axial length and changes in refraction in the NC group. Linear regression line with 95% confidence intervals is shown. **d** Changes in choroidal thickness expressed as interocular differences in the three groups. **e** Changes in choroidal blood perfusion (relative units) expressed as interocular differences in the three groups. **f** Correlation between changes in choroidal thickness and choroidal blood perfusion across all stimulation conditions. Linear regression lines with 95% confidence intervals are shown for each group. Data presentation: All data in (**a**, **b**, **d**, and **e**) are presented as scatter plots with mean ± SEM. Statistical analyses: One-way ANOVA with Tukey’s post-hoc test for (**a**, **b**, **d**, **e**); Pearson correlation for (**c**) and (**f**). *P < 0.05, **P < 0.01, ***P < 0.001. NC, normal control; SEM, standard error of the mean; n = 21 animals per group (interocular differences were calculated from paired eyes of the same animal)

After seven days of lens wear, all eyes treated with − 4.0 D lenses developed significant myopia compared to their respective contralateral control eyes (Table 1). At baseline, no significant differences were observed among the groups for any parameter in either eye (all *P* > 0.05). Paired t-tests also confirmed interocular symmetry within each group (all *P* > 0.05), indicating successful randomization and comparable baseline conditions across all experimental groups (Table 1). To isolate the net treatment effect and control for inter-individual variability, we calculated interocular differences for all parameters as Δ = change in lens-treated right eye − change in untreated left eye. Refractive changes differed significantly among stimulation groups. The ON group showed a significantly smaller myopic shift (Δrefraction: − 2.13 ± 0.40 D) compared to both the NC group (− 4.03 ± 0.35 D, mean difference: 1.90 D, 95% CI: 0.46 to 3.34 D, *P* = 0.0068) and the OFF group (− 3.98 ± 0.50 D, mean difference: 1.85 D, 95% CI: 0.41 to 3.29 D, *P* = 0.0086) (Fig. 1a). No significant difference was observed between the NC and OFF groups (*P* = 0.9959), indicating that OFF stimulation did not influence refractive development. No significant differences in AL were detected among groups; ΔAL in the ON group was comparable to that in the NC and OFF groups (0.085 ± 0.013 mm vs. 0.087 ± 0.018 mm and 0.093 ± 0.034 mm, respectively; both *P* > 0.97) (Fig. 1b). Despite the lack of group differences, Pearson’s correlation analysis revealed a moderately negative correlation between refractive and AL changes in the NC group (R^2^ = 0.366; *P* = 0.0037) (Fig. 1c), consistent with the expected relationship between myopia and axial elongation.

**Table 1:** Ocular parameters at baseline and after 7 days of visual stimulation

Parameter	Group	Day 0		Day 7	
		OD	OS	OD	OS
Refraction (D)	ON	5.90 ± 0.28	5.50 ± 0.29	3.83 ± 0.37	5.56 ± 0.25
	NC	5.44 ± 0.33	5.28 ± 0.40	1.26 ± 0.43	5.13 ± 0.36
	OFF	5.38 ± 0.29	5.36 ± 0.33	0.83 ± 0.50	4.79 ± 0.61
Axial length (mm)	ON	7.78 ± 0.02	7.76 ± 0.02	7.89 ± 0.03	7.80 ± 0.02
	NC	7.82 ± 0.02	7.81 ± 0.02	8.03 ± 0.03	7.93 ± 0.03
	OFF	7.81 ± 0.02	7.82 ± 0.02	8.03 ± 0.03	7.98 ± 0.03
ChT (μm)	ON	68.37 ± 2.48	65.92 ± 3.07	60.51 ± 2.47	66.54 ± 3.16
	NC	67.95 ± 2.28	63.81 ± 1.41	59.99 ± 1.97	67.85 ± 2.44
	OFF	66.59 ± 2.42	68.44 ± 2.20	63.23 ± 1.79	68.14 ± 2.64
ChBP (%)	ON	37.17 ± 1.72	34.15 ± 2.17	33.03 ± 1.42	36.29 ± 1.80
	NC	39.10 ± 1.12	35.40 ± 1.31	33.89 ± 1.06	38.89 ± 1.65
	OFF	35.70 ± 1.19	36.94 ± 1.31	33.70 ± 0.82	34.42 ± 2.24

Data are presented as mean ± SEM. *OD* = right eye (lens-treated); *OS* = left eye (untreated control); *ChT* = choroidal thickness; *ChBP* = choroidal blood perfusion; *NC* = normal control; *SEM* standard error of the mean; n = 21 animals (data from both eyes of the same animal were used for paired comparison)

ChT also differed significantly among the groups. The OFF group exhibited significantly less choroidal thinning than the NC group (− 3.06 ± 2.23 μm vs. − 12.35 ± 2.70 μm; mean difference: − 9.29 μm; 95% CI: − 17.18 to − 1.41 μm; *P* = 0.0171) (Fig. 1d). The ON group showed an intermediate value (− 7.94 ± 1.98 μm) that did not differ significantly from either NC (*P* = 0.3758) or OFF (*P* = 0.3047). For ChBP, OFF stimulation was associated with preservation of choroidal perfusion. The OFF group exhibited a positive Δvalue (0.51 ± 2.18), indicating relative preservation of perfusion in lens-treated eyes (Fig. 1e). This was significantly different from both the ON group (− 6.11 ± 1.76, mean difference: − 6.63, 95% CI: − 12.81 to − 0.44, *P* = 0.0330) and the NC group (− 7.78 ± 1.44, mean difference: − 8.29, 95% CI: − 14.48 to − 2.11, *P* = 0.0057). No significant difference was observed between the ON and NC groups (*P* = 0.7942). Pearson’s correlation analysis revealed a significant positive correlation between ChT and ChBP changes across all stimulation conditions (ON: R^2^ = 0.548, *P* = 0.0001; NC: R^2^ = 0.600, *P* < 0.0001; OFF: R^2^ = 0.727, *P* < 0.0001) (Fig. 1f), confirming the tight coupling between choroidal structure and vascular function.

### The levels of DA and its metabolites vary as a function of visual stimulation-induced refractive development in myopia (with LIM)

**Fig. 2:**
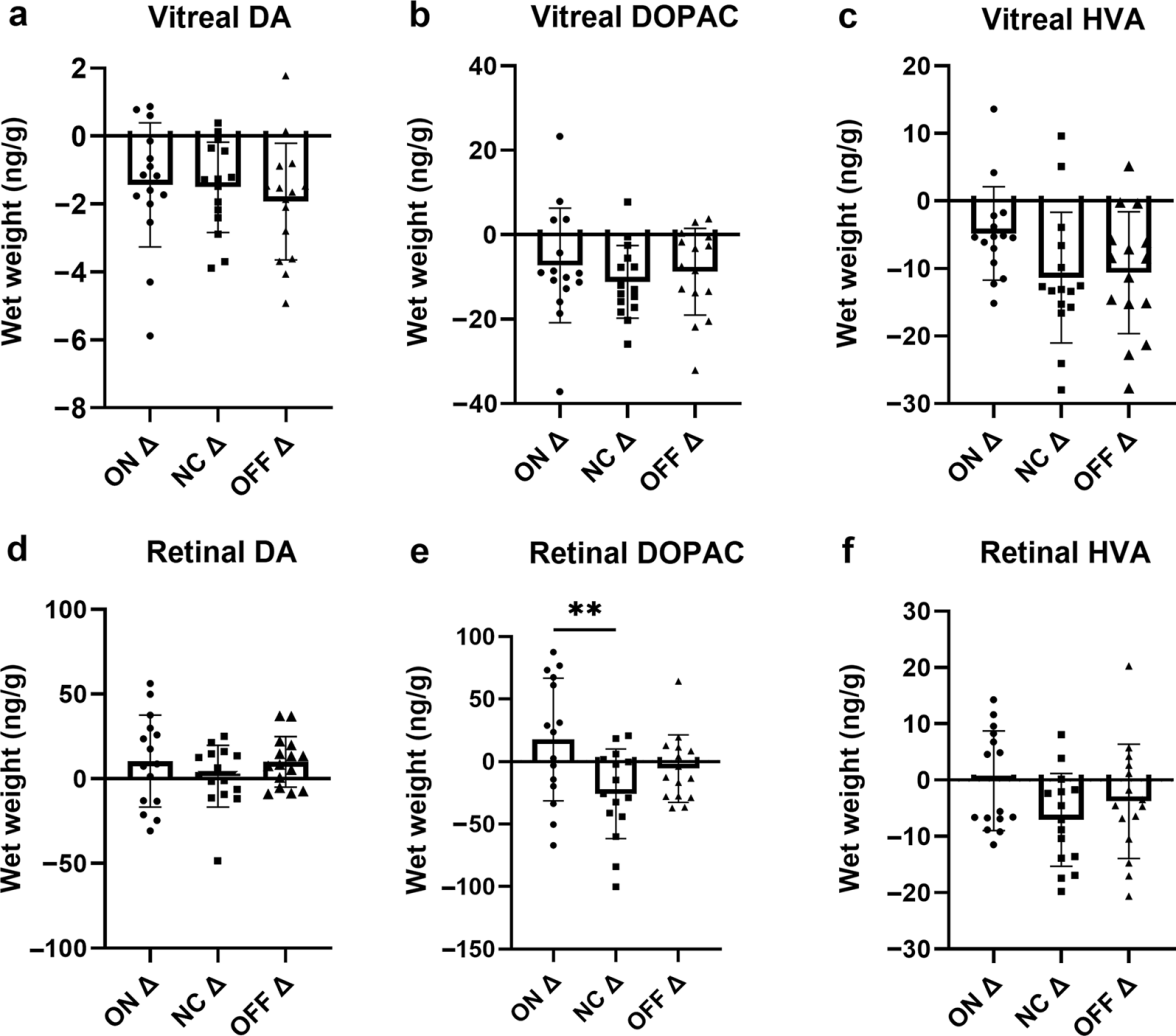
Retinal and vitreal dopamine (DA) and metabolite levels in response to ON and OFF stimulation. Interocular differences (Δ = lens-treated right eye − untreated left eye) in vitreal and retinal DA and its metabolites 3,4-dihydroxyphenylacetic acid (DOPAC) and homovanillic acid (HVA) after 7 days of ON, OFF, or normal control (NC) stimulation with − 4.0 D lenses. Data are presented as scatter plots with mean ± SEM. **a** Vitreal DA levels. **b** Vitreal DOPAC levels. **c** Vitreal HVA levels. **d** Retinal DA levels. **e** Retinal DOPAC levels. **f** Retinal HVA levels. Statistical analyses: One-way ANOVA with Tukey’s post-hoc test was used for all panels. SEM, standard error of the mean. *P < 0.05, **P < 0.01. n = 21 per group for all panels.

To explore the underlying mechanism, we measured DA and its metabolites in the vitreous humor and retina. Interocular differences (Δ) were calculated as described above. Vitreal DA and its metabolites showed no statistically significant differences among groups. Although ON stimulation tended to preserve homovanillic acid (HVA) levels compared with NC and OFF stimulation, this difference was not statistically significant (− 4.82 ± 1.79 vs. − 11.35 ± 2.49 and − 10.63 ± 2.33 ng/g wet weight, respectively; ON vs. NC: *P* = 0.1069; ON vs. OFF: *P* = 0.1671; Fig. 2c).

In contrast, retinal dopaminergic markers revealed significant group differences, particularly for 3,4-dihydroxyphenylacetic acid (DOPAC). Tukey’s post-hoc analysis revealed that the ON group exhibited significantly higher DOPAC levels (ΔDOPAC: 17.65 ± 12.67 ng/g wet weight) compared to the NC group (− 25.75 ± 9.23 ng/g wet weight), with a mean difference of 43.40 ng/g wet weight (*P* = 0.0095) (Fig. 2e). The OFF group showed intermediate values (− 5.59 ± 6.95 ng/g wet weight) that did not differ significantly from either ON (*P* = 0.2324) or NC (*P* = 0.3302).

For retinal DA, both ON (ΔDA: 10.39 ± 7.02 ng/g wet weight) and OFF (ΔDA: 10.00 ± 3.85 ng/g wet weight) stimulation elevated levels compared to NC (1.49 ± 4.71 ng/g wet weight), but these differences were not statistically significant (ON vs. NC: *P* = 0.4750; OFF vs. NC: *P* = 0.5060) (Fig. 2d). Similarly, retinal HVA showed a trend toward preservation in the ON group (ΔHVA: − 0.12 ± 2.28 ng/g wet weight) compared to NC (− 7.09 ± 2.13 ng/g wet weight) and OFF (− 3.77 ± 2.62 ng/g wet weight), but this did not reach statistical significance (ON vs. NC: *P* = 0.1034; ON vs. OFF: *P* = 0.5214) (Fig. 2f).

### Differences in changes in choroidal proteins between the two visual stimulations

#### DEP identification across the three groups with different visual stimulations

**Fig. 3:**
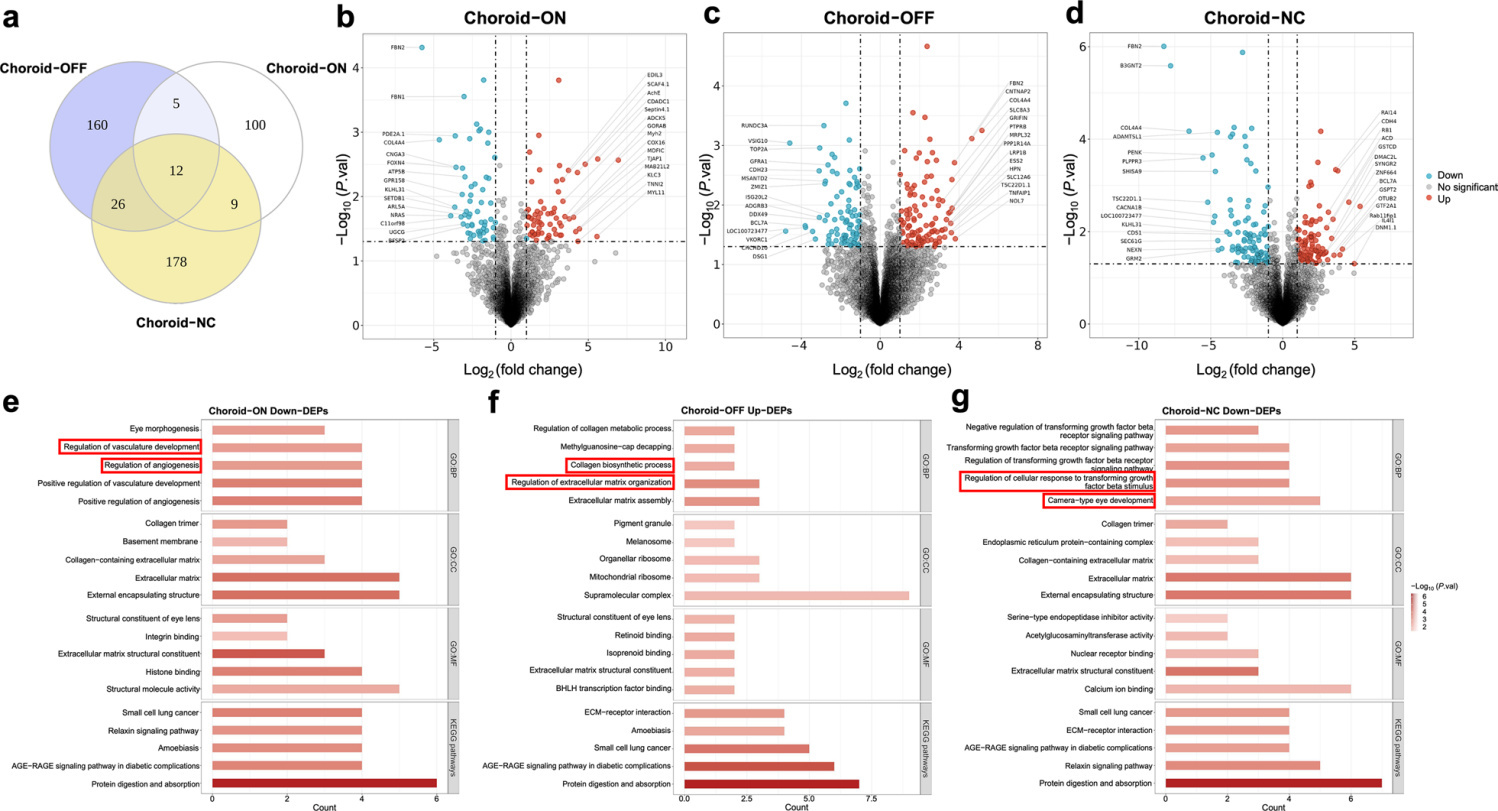
Differentially expressed proteins (DEPs) in the indicated groups. Gene ontology (GO) annotation and Kyoto Encyclopedia of Gene and Genomes (KEGG) pathway enrichment analysis of the DEPs in the indicated stimulus groups. The enriched GO functional annotations were divided into biological process (BP), molecular function (MF), and cellular component (CC): **a** 490 DEPs were identified in all groups. 126 DEPs were identified in the ON group, 203 DEPs were identified in the OFF group, and 225 DEPs were identified in the normal control (NC) group; **b**–**d** Volcano plot showing DEPs that were differentially expressed between the myopic and control eyes under different light stimulation and illustration of the top 15 DEPs that were up or down regulated. Red and blue dots represent significant DEPs (fold changes > 2 or < 0.5 and *P* < 0.05), and the gray dots denote proteins that were not differently expressed in both eyes; **e**–**g** the KEGG enrichment pathways analysis revealed that enriched pathways were associated with protein digestion and absorption and AGE–RAGE signaling pathway in diabetic complications; **e** In the ON Down-DEPs, positive regulation of angiogenesis and vascular development in BP was significantly enriched; **f** In the OFF Up-DEPs, extracellular matrix assembly, regulation of extracellular matrix organization, and collagen biosynthetic process were the most important BPs; **g** In the NC Down-DEPs, significant enrichment was observed in camera-type eye development and regulation of cellular response to transforming growth factor beta stimulus of BP

The choroids of both the myopic and control eyes were categorized into three groups: ON, OFF, and NC. Comprehensive analysis of all choroidal samples revealed 8306 proteins. After inducing myopia under the ON, OFF, and NC conditions, 490 proteins were differentially expressed, with distinct DEP profiles observed in each group (Fig. 3a). Specifically, the ON group had 126 DEPs, including 67 upregulated (Up-DEPs) and 59 downregulated DEPs (Down-DEPs) (Fig. 3b, Table S1). Within the OFF group, 203 DEPs were identified, of which 112 and 91 were Up-DEPs and Down-DEPs, respectively (Fig. 3c, Table S1). In the NC group, 225 DEPs were identified, of which 108 and 117 were Up-DEPs and Down-DEPs, respectively (Fig. 3d, Table S1). The top 15 Up-DEPs and Down-DEPs were visualized using volcano plots (Fig. 3b–d) and heat maps (Fig. S1). In the ON group, Fibrillin 2 (FBN2; log_2_ [FC] = − 5.76, *P* < 0.001), collagen type IV alpha 4 chain (COL4A4; log_2_ [FC] = − 4.64, *P* = 0.001), and GTPase NRas (log_2_ [FC] = − 3.90, *P* = 0.020) were significantly downregulated (Fig. S1). Conversely, in the OFF group, COL4A4 (log_2_ [FC] = 5.16, *P* < 0.001), Contactin Associated Protein Like 2 (log_2_ [FC] = 4.65, *P* < 0.001), Nucleolar Protein 7 (NOL7, log_2_ [FC] = 3.81, *P* = 0.037), and FBN2 (log_2_ [FC] = 3.77, *P* = 0.002) were significantly upregulated (Fig. S1). In the NC group, FBN2 (log_2_ [FC] = − 8.26, *P* < 0.001) exhibited the lowest expression, followed by hexosyltransferase (B3GNT2, log_2_ (FC) = − 7.79, *P* < 0.001) and COL4A4 (log_2_ [FC] = − 6.51, *P* < 0.001) (Fig. S1).

#### Choroidal collagen differences between visual stimulations

DEPs in the ON/OFF/NC groups were further categorized based on GO and KEGG enrichment terms. Across all groups, the most significantly enriched GO cellular-component terms were the external encapsulating structure and the extracellular matrix (ECM). Notably, variations were observed in the most significant BP and molecular functions values across the groups (Fig. S2). In contrast, ON and OFF choroidal DEPs were enriched in similar KEGG pathways, including protein digestion and absorption and the AGE-RAGE signaling pathway in diabetic complications (Fig. S2a–b). Furthermore, the KEGG enrichment pathways in the NC group included focal adhesion, protein digestion, and absorption (Fig. S2c).

**Fig. 4:**
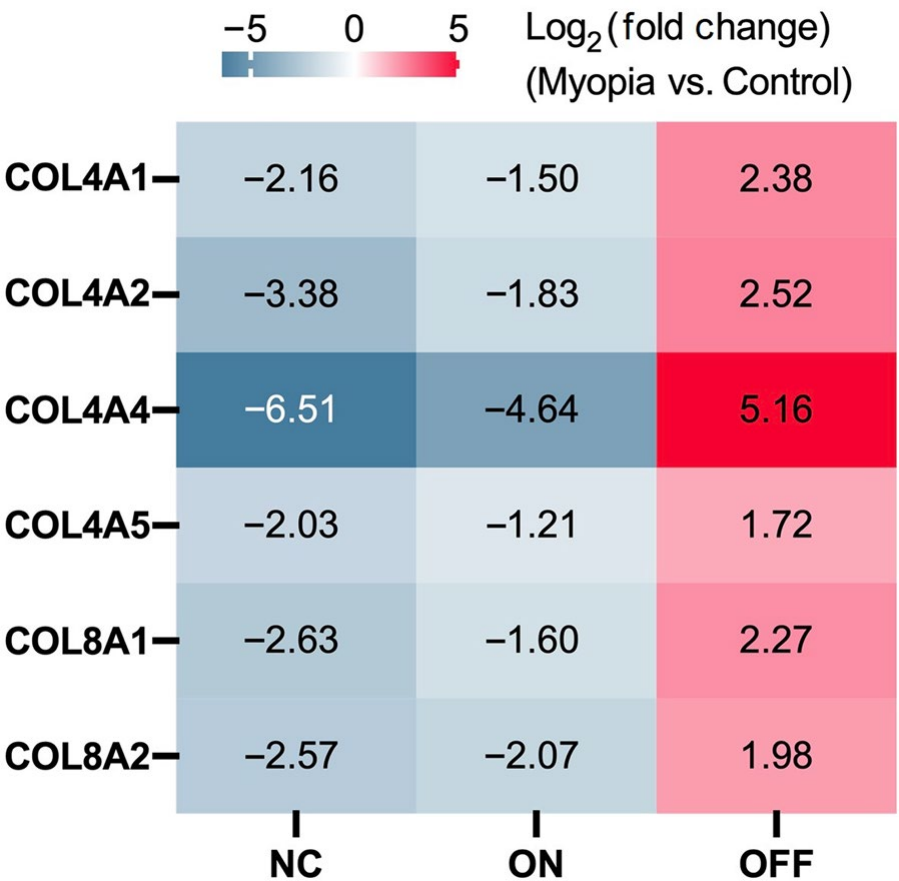
Heatmap showing the significant Kyoto Encyclopedia of Genes and Genomes (KEGG) pathways associated with choroidal collagen expression per group. The box color represents the log_2_ (fold changes) of collagen proteins. NC, normal control

Enrichment analyses of Up-DEPs and Down-DEPs in each group revealed distinct patterns. In the ON and NC groups, enriched pathways were driven primarily by Down-DEPs, whereas in the OFF group they were driven mainly by Up-DEPs. In contrast, they were primarily involved in the Up-DEPs in the OFF group (Fig. 3e–g). In the ON group, the crucial BPs associated with ON Down-DEPs included the positive regulation of angiogenesis and vascular development (Fig. 3e). Conversely, in the OFF group, BPs such as ECM assembly, ECM organization regulation, and collagen biosynthetic processes were prominent among OFF Up-DEPs (Fig. 3f). In the NC group, Down-DEPs were enriched in BPs such as camera-type eye development and regulation of cellular response to transforming growth factor beta stimulus (Fig. 3g). Figure 3 depicts the KEGG enrichment pathways for all three groups, showing notable pathways, including protein digestion and absorption and the AGE-RAGE signaling pathway in diabetic complications. Table S2 details the DEPs associated with these two pathways in each group, highlighting the involvement of type IV and type VIII collagens. Furthermore, a heat map depicting the differential expression of collagen across the three groups revealed relatively higher expression levels in the choroid tissue of myopic eyes in the ON and OFF groups than in the NC group (Fig. 4).

## Discussion

In this study, we investigated how ON and OFF visual stimulation modulate refractive development, AL, ChT, ChBP, choroidal proteomic profiles, and retinal DA metabolism in a guinea pig model of LIM. The principal findings emerged. First, ON stimulation selectively attenuated myopic refractive shift, while OFF stimulation selectively preserved choroidal homeostasis, revealing pathway-specific regulation of ocular responses to myopia. Second, ON stimulation significantly increased retinal DOPAC levels, supporting pathway-specific dopaminergic regulation during LIM. Third, although each visual stimulation generated different DEPs, it was noteworthy that ON and OFF stimulation partially counteracted choroidal collagen type IV (COL4), collagen type VIII (COL8), and FBN2 downregulation under LIM.

Previous studies have implicated the ON pathway in myopia regulation. Crewther et al. reported that additional exposure to ON stimuli reduced LIM in chicks [28]. Conversely, other studies have reported that ON pathway defect mutations in mice may reduce retinal DA metabolism and turnover and increase susceptibility [29–31]. These findings suggest that an intact ON pathway and appropriate DA signaling are crucial for restraining myopic eye growth. However, the relative contributions of ON and OFF stimulation to mammalian myopia development remain insufficiently defined.

Our study demonstrates that ON and OFF stimulation exerted distinct effects on the development of myopia. ON stimulation significantly reduced the myopic refractive shift compared with both the NC and OFF groups. The refractive change in the ON group was notably smaller than that of the NC group (− 4.027 ± 0.352 D) and the OFF group (− 3.975 ± 0.504 D), indicating a refractive protective effect of ON stimulation. However, no significant differences in AL were observed among the groups, suggesting that the observed refractive protection may have been mediated by non-axial mechanisms, such as corneal or lenticular changes. Interestingly, OFF stimulation did not exhibit a similar refractive protective effect, but preserved choroidal integrity, suggesting that OFF stimulation may primarily modulate choroidal homeostasis, while ON stimulation primarily regulated refractive development.

These findings align with previous research. ON stimulation has been linked to refractive protection, as reported by Aung et al. Short-term reading of bright text on dark backgrounds and artificial dynamic ON stimulation have been shown to promote choroidal thickening in humans and chickens [7, 11]. Furthermore, the short-term reading of text with a specific contrast polarity, thought to preferentially stimulate ON pathways, is associated with AL shortening [10]. Our study extends these observations by showing that ON stimulation was associated with preservation of ChBP and a more stable choroidal environment, potentially contributing to its protective effect against myopia progression. Numerous studies have reported choroidal thinning in myopic patients [32], and observational studies have indicated that ChT may influence AL growth and ChBP, thus helping predict the risk of myopia [14]. Increased ChBP inhibits the progression of deprivation myopia in guinea pigs [33]. Human data indicate that preferential OFF-pathway stimulation produces greater accommodation-induced choroidal thinning than ON-biased stimulation [6]. In the present study, OFF stimulation induced compensatory upregulation of collagen and fibrillin, suggesting structural protection of the choroid despite the absence of refractive protection. In chickens, 7 day-ON/OFF stimulation showed no statistically significant effects on ChT, either in myopic eyes or control eyes [11], suggesting that species- or protocol-specific factors may modulate the choroidal response. The ON group maintained a relatively stable choroidal profile, whereas OFF stimulation produced pronounced ECM upregulation, indicating distinct adaptive remodeling responses. These choroidal findings further support the differential effects of ON and OFF stimulation. ON stimulation may help maintain choroidal homeostasis, whereas OFF stimulation may drive adaptive changes in the choroidal ECM, without producing comparable refractive control.

DA metabolism plays a key role in mediating ON/OFF-dependent ocular responses. In chickens, flickering light (1 Hz) at 20 lx increases retinal DA and DOPAC release, compared to 470 lx, while reducing vitreal DA and DOPAC release [34]. Artificial dynamic ON stimulation has been shown to increase both retinal and vitreal DA [13]. Both ON and OFF stimulation produced higher retinal DA levels compared to NC conditions, but only the ON group showed a significant increase in retinal DOPAC levels. This pattern suggests that ON stimulation enhances retinal DA turnover rather than merely increasing DA availability, thereby creating a biochemical milieu that may stabilize ocular growth. The concept that ON pathway activation helps maintain a more physiological and homeostatic state of retinal DA signaling is supported by several recent studies. Aung et al. showed that genetic disruption of ON pathways in mice led to markedly reduced retinal DA and DOPAC levels and impaired DA turnover, whereas the partial loss of OFF pathway function had only minimal effects on dopaminergic signaling [29]. Landis et al. further demonstrated that ambient light levels modulate DA-related genes and proteins and interact with lens defocus to alter myopia susceptibility, highlighting the importance of light-driven ON pathway activity in normal dopaminergic regulation [35]. Consistent with this, Shu et al. identified retinal DA D1 receptors, predominantly linked to ON-pathway signaling, as critical mediators of dopaminergic antimyopic effects in FDM [36]. Together with recent reviews on light signaling and myopia [37, 38], these results support our interpretation that effective ON pathway activation helps preserve retinal DA signaling, thereby limiting the biochemical conditions that favor excessive axial elongation.

It should be noted that dopaminergic markers were assessed after 7 days of lens wear, refractive compensation was likely already established. This time point likely reflects a relatively steady-state condition, whereas earlier sampling may reveal distinct response dynamics, consistent with reports of time-dependent dopaminergic changes under visual stimulation [39]. Therefore, future studies should characterize the temporal profile of DA metabolism during the early phases of myopia induction.

In summary, this study demonstrated that ON and OFF stimulation exert differential effects on myopia development. ON stimulation was primarily associated with attenuation of refractive progression, potentially through enhanced retinal DA metabolism, whereas OFF stimulation was more closely linked to preservation of choroidal homeostasis.

At the choroidal proteomic level, our findings indicate that ON stimulation limited LIM-induced remodeling and preserved a more homeostatic molecular profile, whereas OFF and NC paradigms were associated with distinct forms of broader proteomic remodeling. Under the same LIM load, the ON group exhibited the smallest number of choroidal DEPs, compared with the more extensive proteomic reprogramming observed in the OFF and NC groups. This restricted molecular response under ON stimulation paralleled the relatively favorable choroidal perfusion observed in our biometric data, suggesting that ON stimulation may allow the choroid to adapt to defocus with minimal disruption of tissue homeostasis.

Notably, key ECM components, including COL4, COL8, and FBN2, were markedly downregulated in the NC group, indicating reduced choroidal matrix support consistent with the most severe myopia and choroidal thinning observed. The NC group represented the baseline response to LIM under conventional laboratory illumination, with the right eye serving as the myopia-induced state rather than an untreated absolute control. In contrast, both ON and OFF groups exhibited protective modulation through distinct mechanisms. ON stimulation promoted moderate, coordinated ECM adjustments compatible with choroidal stability, whereas OFF stimulation induced marked upregulation of collagen and fibrillin, suggesting compensatory matrix deposition response. Both patterns were associated with improved ChT and ChBP, indicating that ON and OFF stimulation may exert protective effects through different pathways.

Collagen, particularly COL4 and COL8, is a major structural component of the ECM and basement membranes and is essential for maintaining vascular-wall and choroidal-tissue integrity [40]. COL4 not only provides mechanical support but also participates in cell adhesion, migration, and angiogenesis regulation, influencing vascular stability [41, 42]. Type VIII collagen comprises α1 (COL8A1) and α2 (COL8A2) collagen chains [43]. COL8 is a member of the short-chain collagen family and is expressed in low amounts in normal arteries [44]. COL8A1 has been detected in the Bruch's membrane, and COL8A1 variants have been implicated in age-related macular degeneration [45]. Both COL4 and COL8 contribute to vascular and ocular homeostasis as well as tissue remodeling.

FBN2 forms the core of microfibrils in the ECM and contributes to tissue stability [46]. FBN2 influences the elasticity of the RPE, choroid, and Bruch’s membrane, and mutations in FBN2 have been linked to early-onset macular degeneration [47, 48]. Our findings suggest that FBN2 downregulation under NC conditions may weaken choroidal and Bruch's membrane elasticity, whereas its moderate reduction under ON stimulation and compensatory upregulation under OFF stimulation may help preserve the mechanical resilience of the posterior pole during LIM.

The choroid, located between the retina and the sclera, is one of the most vascularized tissues in the body. In this study, COL4, COL8, and FBN2 showed distinct expression patterns across the three visual-stimulation conditions. As shown in Fig. 3 and Fig. S1, these proteins were strongly downregulated in the myopic choroid of the NC group, moderately reduced in the ON group, and markedly upregulated in the OFF group. Despite the lack of refractive protection, the OFF group exhibited preserved ChT and ChBP, accompanied by a marked upregulation of COL4, COL8, and FBN2. This suggests that choroidal protection can occur independently without DA metabolic support (as evidenced by the unchanged DOPAC levels). The over-compensatory increase in collagen and fibrillin may represents an active ECM remodeling response that maintains choroidal structural integrity even in the absence of a refractive benefit. These findings suggest that choroidal protection is not necessarily coupled to refractive outcomes and can be achieved through ECM-mediated mechanisms alone, highlighting the unique role of the OFF pathway in preserving choroidal structure and vascular function under myopiagenic stress.

### Shortcomings and prospects

This study has some limitations. First, fellow eye control is not truly independent, as it may exhibit consensual growth responses to monocular defocus via central mechanisms, potentially leading to conservative estimates of treatment effects. Second, ON- and OFF-pathway activation or inhibition was not directly validated. While the sawtooth stimuli were designed to bias ON or OFF activity, they did not achieve complete pathway isolation owing to functional coupling between ON and OFF circuits. Thus, the observed effects should be interpreted as responses to differences in stimulus statistics rather than as evidence of fully selective pathway manipulation. Future studies should employ electrophysiological validation (e.g., scotopic threshold response [STR]/photopic negative response [PhNR] for the ON-pathway, oscillatory potentials [OPs] for the OFF-pathway), pharmacological manipulations, and flicker controls matched for temporal frequency and contrast energy to confirm pathway-specific effects. Third, the molecular mechanisms of ChT and ChBP changes, choroidal protein changes, and retinal DA changes were not determined in the present study. Fourth, because of species differences between rodents and humans, these findings cannot be directly extrapolated to humans; validation in primate models may be required before translational application.

Our findings suggest that artificial dynamic ON and OFF visual stimulation may differentially affect myopia development in guinea pigs by modulating choroidal structure, choroidal perfusion, and retinal DA metabolism. Specifically, ON pathway may reduce the risk or progression of myopia in guinea pigs, providing mechanistic insights into refractive development across experimental models. With further optimization and validation, dynamic visual stimuli may offer a screen-based strategy to slow myopia progression during reading or near-work activities.

## Conclusion

This study indicates that artificial dynamic ON and OFF stimulation can modify refraction, AL, ChBP, choroidal collagen and fibrillin expression, and DA release from the retina. Further research is necessary to confirm the relationships between these changes and to explore potential strategies for optimizing and enhancing the positive effects.

## Supplementary Information

Additional file1 (DOCX 742 KB)

## Data Availability

In this study, the original contributions are included in the article, and further inquiries can contact the corresponding author directly.

## Abbreviations

ChT: Choroidal thickness
AL: Axial length
DA: Dopamine
OCT: Optical coherence tomography
OCTA: OCT angiography
ChBP: Choroidal blood perfusion
RPE: Retinal pigment epithelium
FDM: Form-deprivation myopia
LIM: Lens-induced myopia
HPLC: High-performance liquid chromatography
LC–MS/MS: Liquid chromatography–mass spectrometry/mass spectrometry
DEPs: Differentially expressed proteins
KEGG: Kyoto Encyclopedia of Genes and Genomes
BPs: Biological processes
DOPAC: 3,4-Dihydroxyphenylacetic acid
HVA: Homovanillic acid
ECM: Extracellular matrix
NO: Nitric oxide
COL4: Collagen type IV
COL8: Collagen type VIII
FBN2: Fibrillin 2
Up-DEPs: Upregulated DEPs
Down-DEPs: Downregulated DEPs
NOL7: Nucleolar protein 7
B3GNT2: Hexosyltransferase
COL8A1: Type VIII collagen comprises α1
COL8A2: Type VIII collagen comprises α2
