# Supplementary material for: Differential regulation of myopia progression by ON and OFF stimulation in guinea pigs
Source: Eye Vis (Lond). 2026 Jun 9;13:24. doi: 10.1186/s40662-026-00495-z (PMC13248434; doi:10.1186/s40662-026-00495-z)
Supplement: Supplementary file 1 — Additional file1 (DOCX 742 KB) [file 40662_2026_495_MOESM1_ESM.docx]

**Supplementary Materials**

**Supplementary Figure S1**. The heatmap of differentially expressed proteins (DEPs) per group. The box color represents the log_2_ (fold changes) of DEPs, transitioning from blue (decreasing) to red (increasing). NC, normal control.

**
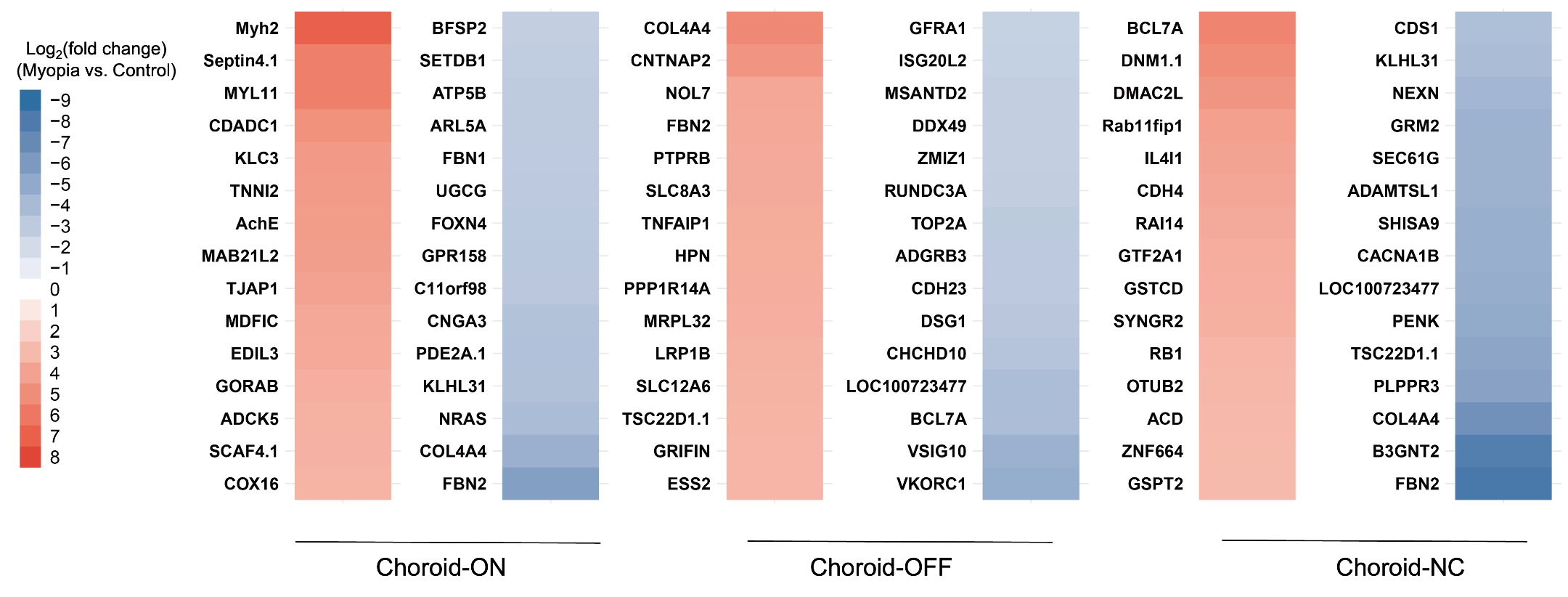
**

**Figure S2**. Gene Ontology (GO) and Kyoto Encyclopedia of Genes and Genomes (KEGG) enrichment analysis of differentially expressed proteins (DEPs) in the three groups. The most significant cellular component (CC) among the groups was the external encapsulating structure. **a** In the ON group, sensory organ development in biological process (BP) and protein digestion and absorption in KEGG were significantly enriched; **b** In the OFF group, extracellular matrix assembly was the most important BP. Protein digestion and absorption was the most significant enriched KEGG pathway; **c** In the normal control (NC) group, eye morphogenesis of BP and focal adhesion of KEGG were enriched


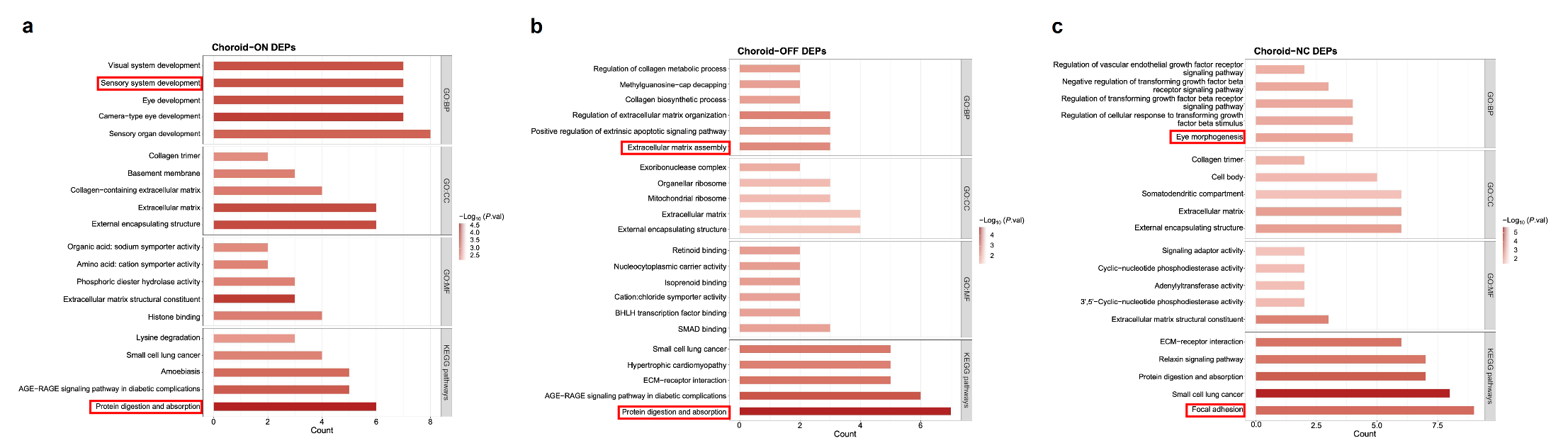


**Figure S3.** Bland–Altman plots for inter-operator agreement of choroidal thickness measurements at pre (Day 0, baseline) and post (Day 7, after lens wear) in right (OD) and left (OS) eyes. Each point represents the difference between Operators A and B plotted against their mean for individual samples (n = 19 per condition). Solid horizontal lines indicate the mean difference (bias); dashed lines represent the 95% limits of agreement (LoA, mean difference ± 1.96 SD). **a** Right eye at Day 0. Bias: −2.08 μm (95% LoA: −5.50 to 1.35 μm). **b** Left eye at Day 0. Bias: −1.38 μm (95% LoA: −5.94 to 3.19 μm). **c** Right eye at Day 7. Bias: −0.98 μm (95% LoA: −5.51 to 3.55 μm). **d** Left eye at Day 7. Bias: −1.14 μm (95% LoA: −5.92 to 3.65 μm).


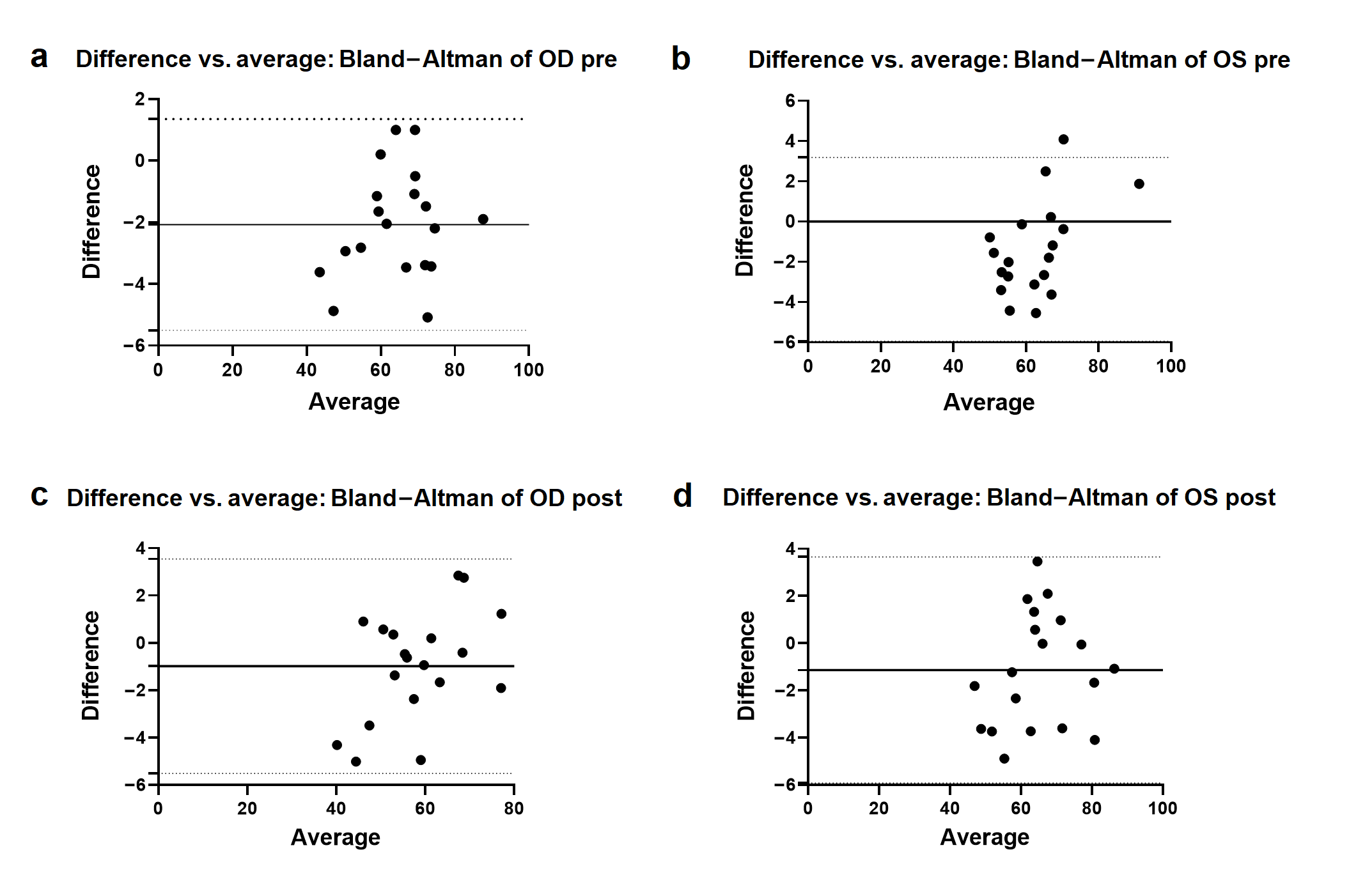


|  | | | | |
| --- | --- | --- | --- | --- |
| **Supplementary Table S1. List of differentially expressed proteins in each visual stimuli group after myopia induction.** | | | | |
| **A. ON** | | | | |
|  | **Gene name** | **Protein name** | **Log_2_ (FC)** | ***P* value** |
| **1** | Myh2 | Myosin heavy chain 2 | 6.94 | 0.002714 |
| **2** | Septin4.1 | Septin-type G domain-containing protein | 5.61 | 0.002607 |
| **3** | MYL11 | Myosin light chain, phosphorylatable, fast skeletal muscle | 5.56 | 0.041728 |
| **4** | CDADC1 | Cytidine and dCMP deaminase domain containing 1 | 4.80 | 0.003139 |
| **5** | KLC3 | Kinesin light chain | 4.43 | 0.031679 |
| **6** | TNNI2 | Troponin I2, fast skeletal type | 4.33 | 0.049599 |
| **7** | AchE | Carboxylic ester hydrolase | 4.30 | 0.004233 |
| **8** | MAB21L2 | Mab-21 like 2 | 4.19 | 0.034975 |
| **9** | TJAP1 | Tight junction associated protein 1 | 4.05 | 0.020933 |
| **10** | MDFIC | MyoD family inhibitor domain containing | 3.77 | 0.022241 |
| **11** | EDIL3 | EGF like repeats and discoidin domains 3 | 3.72 | 0.003920 |
| **12** | GORAB | RAB6-interacting golgin | 3.49 | 0.020462 |
| **13** | ADCK5 | AarF domain containing kinase 5 | 3.36 | 0.011823 |
| **14** | SCAF4.1 | SR-related CTD associated factor 4 | 3.33 | 0.005464 |
| **15** | COX16 | Cytochrome c oxidase assembly protein COX16 homolog, mitochondrial | 3.25 | 0.028558 |
| **16** | PAN3 | PAN2-PAN3 deadenylation complex subunit PAN3 | 3.25 | 0.039768 |
| **17** | TSC22D1.1 | TSC22 domain family member 1 | 3.17 | 0.039827 |
| **18** | MARCHF6 | Membrane associated ring-CH-type finger 6 | 3.15 | 0.025697 |
| **19** | KRT14 | Keratin 14 | 3.14 | 0.003297 |
| **20** | MPP4 | Membrane palmitoylated protein 4 | 3.09 | 0.000156 |
| **21** | SLC6A15 | Transporter | 3.07 | 0.008447 |
| **22** | C5orf22 | Chromosome 5 open reading frame 22 | 2.95 | 0.005770 |
| **23** | SLC1A3 | Amino acid transporter | 2.93 | 0.021671 |
| **24** | GRIK1 | Glutamate receptor | 2.90 | 0.038177 |
| **25** | PLCD4 | Phosphoinositide phospholipase C | 2.81 | 0.015234 |
| **26** | HOMER2 | Homer scaffold protein 2 | 2.80 | 0.032361 |
| **27** | UNC13D | Unc-13 homolog D | 2.75 | 0.017606 |
| **28** | ISOC2 | Isochorismatase domain containing 2 | 2.61 | 0.018217 |
| **29** | JAG1 | Delta-like protein | 2.55 | 0.048821 |
| **30** | PLEKHB1 | Pleckstrin homology domain containing B1 | 2.53 | 0.026977 |
| **31** | CNTNAP2 | Contactin associated protein like 2 | 2.45 | 0.040220 |
| **32** | CAMKV | CaM kinase like vesicle associated | 2.43 | 0.043806 |
| **33** | RAB43 | RAB43, member RAS oncogene family | 2.39 | 0.018496 |
| **34** | CPEB3 | Cytoplasmic polyadenylation element binding protein 3 | 2.34 | 0.045338 |
| **35** | Sec24c.1 | SEC24 homolog C, COPII coat complex component | 2.13 | 0.015327 |
| **36** | EEPD1 | Endonuclease/exonuclease/phosphatase family domain-containing protein 1 | 1.99 | 0.027082 |
| **37** | RTBDN | Retbindin | 1.99 | 0.036899 |
| **38** | TMEM72 | Transmembrane protein 72 | 1.92 | 0.012015 |
| **39** | INTS8 | Integrator complex subunit 8 | 1.91 | 0.018090 |
| **40** | CLNS1A | Methylosome subunit pICln | 1.87 | 0.025302 |
| **41** | SPAST | Spastin | 1.85 | 0.003845 |
| **42** | SDSL | L-serine ammonia-lyase | 1.82 | 0.030785 |
| **43** | QPRT | Nicotinate-nucleotide pyrophosphorylase [carboxylating] | 1.80 | 0.001118 |
| **44** | GJC3 | Gap junction protein | 1.77 | 0.021195 |
| **45** | NSD3 | Nuclear receptor binding SET domain protein 3 | 1.77 | 0.036030 |
| **46** | RBCK1 | RanBP-type and C3HC4-type zinc finger-containing protein 1 | 1.75 | 0.024934 |
| **47** | TUBG2 | Tubulin gamma chain | 1.75 | 0.038503 |
| **48** | RUNDC3A | RUN domain containing 3A | 1.70 | 0.016951 |
| **49** | RSBN1L | Round spermatid basic protein 1 like | 1.64 | 0.047638 |
| **50** | MED12 | Mediator complex subunit 12 | 1.57 | 0.019037 |
| **51** | GDPD5 | Glycerophosphodiester phosphodiesterase domain containing 5 | 1.55 | 0.038851 |
| **52** | DDX55 | ATP-dependent RNA helicase | 1.52 | 0.038150 |
| **53** | EPHB2 | receptor protein-tyrosine kinase | 1.48 | 0.023336 |
| **54** | ZNF503 | Zinc finger protein 503 | 1.46 | 0.027742 |
| **55** | TMTC1 | dolichyl-phosphate-mannose--protein mannosyltransferase | 1.41 | 0.040821 |
| **56** | TMEM9B | TMEM9 domain family member B | 1.38 | 0.035451 |
| **57** | INTS1 | Integrator complex subunit 1 | 1.36 | 0.035731 |
| **58** | NOC4L | Nucleolar complex associated 4 homolog | 1.34 | 0.005865 |
| **59** | LRCH4.1 | Leucine rich repeats and calponin homology domain containing 4 | 1.32 | 0.021925 |
| **60** | MAPKAP1 | Target of rapamycin complex 2 subunit MAPKAP1 | 1.24 | 0.038915 |
| **61** | ITGAM | Integrin subunit alpha M | 1.20 | 0.002037 |
| **62** | CAPS | Calcyphosine | 1.16 | 0.020852 |
| **63** | HPS3 | Hermansky-Pudlak syndrome 3 protein homolog | 1.14 | 0.020706 |
| **64** | ANAPC1 | Anaphase promoting complex subunit 1 | 1.12 | 0.015871 |
| **65** | ADCK1 | AarF domain containing kinase 1 | 1.10 | 0.020701 |
| **66** | SENP7 | SUMO specific peptidase 7 | 1.04 | 0.028074 |
| **67** | MEMO1 | Protein MEMO1 | 1.01 | 0.017084 |
| **68** | SCARA5 | Scavenger receptor class A member 5 | 1.00 | 0.044793 |
| **69** | ENG | Endoglin | −1.01 | 0.025266 |
| **70** | TSFM | Elongation factor Ts, mitochondrial | −1.05 | 0.002464 |
| **71** | TRIM24 | Tripartite motif containing 24 | −1.12 | 0.045583 |
| **72** | MCM2 | DNA replication licensing factor MCM2 | −1.14 | 0.030362 |
| **73** | INTS13 | Integrator complex subunit 13 | −1.14 | 0.042763 |
| **74** | CD1D | CD1d molecule | −1.30 | 0.022537 |
| **75** | CYB5R4 | Cytochrome b5 reductase 4 | −1.31 | 0.012605 |
| **76** | CD34 | CD34 molecule | −1.44 | 0.001000 |
| **77** | MAGT1 | Magnesium transporter 1 | −1.44 | 0.005219 |
| **78** | COL4A1 | Collagen type IV alpha 1 chain | −1.50 | 0.001476 |
| **79** | GET1 | Guided entry of tail-anchored proteins factor 1 | −1.59 | 0.033647 |
| **80** | COL8A1 | Collagen type VIII alpha 1 chain | −1.60 | 0.005020 |
| **81** | USP3 | Ubiquitin carboxyl-terminal hydrolase | −1.68 | 0.024889 |
| **82** | DENND5A | DENN domain containing 5A | −1.68 | 0.047904 |
| **83** | ASB3 | Ankyrin repeat and SOCS box containing 3 | −1.71 | 0.027877 |
| **84** | Col10a1 | C1q domain-containing protein | −1.77 | 0.031338 |
| **85** | Crybb1 | BetaB1-crystallin | −1.77 | 0.036431 |
| **86** | EMILIN1 | Elastin microfibril interfacer 1 | −1.77 | 0.000155 |
| **87** | YEATS4 | YEATS domain containing 4 | −1.82 | 0.048913 |
| **88** | COL4A2 | Collagen type IV alpha 2 chain | −1.83 | 0.007810 |
| **89** | FBXL18 | F-box and leucine rich repeat protein 18 | −1.92 | 0.025112 |
| **90** | SERINC3 | Serine incorporator 3 | −1.92 | 0.012370 |
| **91** | RBM22 | Pre-mRNA-splicing factor RBM22 | −1.96 | 0.034579 |
| **92** | LTBP1 | Latent transforming growth factor beta binding protein 1 | −1.98 | 0.000895 |
| **93** | ANKS1B | Ankyrin repeat and sterile alpha motif domain containing 1B | −2.01 | 0.041000 |
| **94** | PLCH1 | Phosphoinositide phospholipase C | −2.04 | 0.037732 |
| **95** | COL8A2 | Collagen type VIII alpha 2 chain | −2.07 | 0.000951 |
| **96** | TBC1D22B | TBC1 domain family member 22B | −2.11 | 0.034829 |
| **97** | MTR | Methionine synthase | −2.17 | 0.017073 |
| **98** | EMILIN2 | Elastin microfibril interfacer 2 | −2.22 | 0.000748 |
| **99** | KDM1B | Lysine demethylase 1B | −2.33 | 0.006378 |
| **100** | KMT5A | [histone H4]-lysine(20) N-methyltransferase | −2.41 | 0.039860 |
| **101** | PDE9A | Phosphodiesterase | −2.52 | 0.028172 |
| **102** | CDR2 | Cerebellar degeneration related protein 2 | −2.53 | 0.027163 |
| **103** | ADAMTSL1 | ADAMTS like 1 | −2.57 | 0.006992 |
| **104** | Elavl4 | ELAV-like protein | −2.60 | 0.009501 |
| **105** | RGS6 | Regulator of G protein signaling 6 | −2.63 | 0.045105 |
| **106** | CTU2 | Cytoplasmic tRNA 2-thiolation protein 2 | −2.64 | 0.037704 |
| **107** | RB1 | RB transcriptional corepressor 1 | −2.65 | 0.011783 |
| **108** | CFAP20 | Cilia and flagella associated protein 20 | −2.66 | 0.018778 |
| **109** | MFAP2 | Microfibril associated protein 2 | −2.67 | 0.001261 |
| **110** | SYT12 | Synaptotagmin 12 | −2.73 | 0.020576 |
| **111** | CELF4 | CUGBP Elav-like family member 4 | −2.78 | 0.036800 |
| **112** | BFSP2 | Beaded filament structural protein 2 | −2.81 | 0.028433 |
| **113** | SETDB1 | SET domain bifurcated histone lysine methyltransferase 1 | −2.89 | 0.016981 |
| **114** | ATP5B | ATP synthase subunit beta (Fragment) | −2.98 | 0.004938 |
| **115** | ARL5A | ADP ribosylation factor like GTPase 5A | −3.00 | 0.019327 |
| **116** | FBN1 | Fibrillin 1 | −3.04 | 0.000279 |
| **117** | UGCG | ceramide glucosyltransferase | −3.09 | 0.026681 |
| **118** | FOXN4 | Forkhead box N4 | −3.14 | 0.003621 |
| **119** | GPR158 | G protein-coupled receptor 158 | −3.16 | 0.009279 |
| **120** | C11orf98 | Chromosome 11 open reading frame 98 | −3.23 | 0.020696 |
| **121** | CNGA3 | Cyclic nucleotide gated channel subunit alpha 3 | −3.56 | 0.003502 |
| **122** | PDE2A.1 | Phosphodiesterase | −3.61 | 0.001140 |
| **123** | KLHL31 | Kelch like family member 31 | −3.64 | 0.014588 |
| **124** | NRAS | GTPase NRas | −3.90 | 0.019923 |
| **125** | COL4A4 | Collagen type IV alpha 4 chain | −4.64 | 0.001310 |
| **126** | FBN2 | Fibrillin 2 | −5.76 | 0.000048 |
|  |  |  |  |  |
|  |  |  |  |  |
| **B. OFF** | | | | |
|  | **Gene name** | **Protein name** | **Log_2_ (FC)** | ***P* value** |
| **1** | COL4A4 | Collagen type IV alpha 4 chain | 5.16 | 0.000559 |
| **2** | CNTNAP2 | Contactin associated protein like 2 | 4.65 | 0.000765 |
| **3** | NOL7 | Nucleolar protein 7 | 3.81 | 0.036992 |
| **4** | FBN2 | Fibrillin 2 | 3.77 | 0.001943 |
| **5** | PTPRB | protein-tyrosine-phosphatase | 3.65 | 0.011309 |
| **6** | SLC8A3 | Solute carrier family 8 member A3 | 3.64 | 0.003744 |
| **7** | TNFAIP1 | TNF alpha induced protein 1 | 3.61 | 0.029172 |
| **8** | HPN | Hepsin | 3.58 | 0.020282 |
| **9** | PPP1R14A | Protein phosphatase 1 regulatory subunit 14 | 3.55 | 0.013142 |
| **10** | MRPL32 | Mitochondrial ribosomal protein L32 | 3.48 | 0.013625 |
| **11** | LRP1B | LDL receptor related protein 1B | 3.36 | 0.017613 |
| **12** | SLC12A6 | Solute carrier family 12 member 6 | 3.29 | 0.025395 |
| **13** | TSC22D1.1 | TSC22 domain family member 1 | 3.27 | 0.034737 |
| **14** | GRIFIN | Galectin | 3.17 | 0.008527 |
| **15** | ESS2 | Ess-2 splicing factor homolog | 3.15 | 0.026504 |
| **16** | TNNC1 | Troponin C1, slow skeletal and cardiac type | 3.13 | 0.026481 |
| **17** | HELZ | Helicase with zinc finger | 3.01 | 0.035777 |
| **18** | BSCL2 | Seipin | 3.00 | 0.041871 |
| **19** | NOCT | Nocturnin | 2.98 | 0.008097 |
| **20** | NPY | Neuropeptide Y | 2.95 | 0.010168 |
| **21** | CEP290 | Centrosomal protein 290 | 2.89 | 0.028298 |
| **22** | PDE2A.1 | Phosphodiesterase | 2.88 | 0.005735 |
| **23** | MMADHC | Metabolism of cobalamin associated D | 2.85 | 0.001774 |
| **24** | Crygn | Crystallin gamma N | 2.84 | 0.044358 |
| **25** | MICAL2 | F-actin monooxygenase | 2.81 | 0.016810 |
| **26** | KCNH6 | Potassium voltage-gated channel subfamily H member 6 | 2.80 | 0.040456 |
| **27** | USF1 | Upstream transcription factor 1 | 2.79 | 0.045848 |
| **28** | IGFBP1 | Insulin-like growth factor-binding protein 1 | 2.76 | 0.049893 |
| **29** | P4HTM | Prolyl 4-hydroxylase, transmembrane | 2.75 | 0.026742 |
| **30** | GCHFR | GTP cyclohydrolase 1 feedback regulatory protein | 2.66 | 0.028189 |
| **31** | SCG2 | Secretogranin-2 | 2.66 | 0.020571 |
| **32** | MFAP2 | Microfibril associated protein 2 | 2.65 | 0.001322 |
| **33** | AIMP1 | Aminoacyl tRNA synthetase complex interacting multifunctional protein 1 | 2.62 | 0.027881 |
| **34** | RRP7A | Ribosomal RNA processing 7 homolog A | 2.62 | 0.007796 |
| **35** | PPFIBP1 | PPFIA binding protein 1 | 2.60 | 0.021558 |
| **36** | CRABP2 | Cellular retinoic acid binding protein 2 | 2.59 | 0.003273 |
| **37** | DLGAP1 | DLG associated protein 1 | 2.59 | 0.038383 |
| **38** | COL4A2 | Collagen type IV alpha 2 chain | 2.52 | 0.000785 |
| **39** | ADGRL4 | Adhesion G protein-coupled receptor L4 | 2.48 | 0.031130 |
| **40** | TAMM41 | Phosphatidate cytidylyltransferase, mitochondrial | 2.42 | 0.039858 |
| **41** | LINGO1 | Leucine rich repeat and Ig domain containing 1 | 2.39 | 0.001868 |
| **42** | COL4A1 | Collagen type IV alpha 1 chain | 2.38 | 0.000022 |
| **43** | AFDN.2 | Afadin, adherens junction formation factor | 2.37 | 0.003336 |
| **44** | SPRYD3 | SPRY domain containing 3 | 2.35 | 0.033395 |
| **45** | PPP1R1A | Protein phosphatase 1 regulatory inhibitor subunit 1A | 2.33 | 0.022144 |
| **46** | COL8A1 | Collagen type VIII alpha 1 chain | 2.27 | 0.000336 |
| **47** | TRIT1 | tRNA dimethylallyltransferase | 2.26 | 0.009875 |
| **48** | C1QL2 | Complement C1q like 2 | 2.25 | 0.004110 |
| **49** | CYSTM1 | Cysteine-rich and transmembrane domain-containing protein 1 | 2.21 | 0.041349 |
| **50** | RGCC | Regulator of cell cycle | 2.20 | 0.036343 |
| **51** | PLAAT3 | Phospholipase A and acyltransferase 3 | 2.14 | 0.007342 |
| **52** | TSPAN15 | Tetraspanin | 2.13 | 0.042733 |
| **53** | LACC1 | Laccase domain containing 1 | 2.12 | 0.039904 |
| **54** | SNX15 | Sorting nexin 15 | 2.08 | 0.048814 |
| **55** | SUPT4H1 | Transcription elongation factor SPT4 | 2.05 | 0.039552 |
| **56** | CDK2AP2 | Cyclin-dependent kinase 2-associated protein | 2.03 | 0.026252 |
| **57** | TELO2 | Telomere length regulation protein TEL2 homolog | 2.01 | 0.015138 |
| **58** | THTPA | Thiamine-triphosphatase | 2.00 | 0.046743 |
| **59** | COL8A2 | Collagen type VIII alpha 2 chain | 1.98 | 0.001336 |
| **60** | RSRC2 | Arginine and serine rich coiled-coil 2 | 1.96 | 0.037070 |
| **61** | RBM48 | RNA-binding protein 48 | 1.95 | 0.033881 |
| **62** | MRPL40 | Mitochondrial ribosomal protein L40 | 1.94 | 0.035828 |
| **63** | LOC100732718 | Cytochrome b-c1 complex subunit 6 | 1.93 | 0.039510 |
| **64** | CTIF | Cap binding complex dependent translation initiation factor | 1.92 | 0.027392 |
| **65** | LTBP1 | Latent transforming growth factor beta binding protein 1 | 1.83 | 0.001627 |
| **66** | TAS1R1 | Taste 1 receptor member 1 | 1.82 | 0.012408 |
| **67** | ZFAND3 | Zinc finger AN1-type containing 3 | 1.82 | 0.049898 |
| **68** | C19orf25 | Chromosome 19 open reading frame 25 | 1.78 | 0.045107 |
| **69** | Krt12 | Keratin 12 | 1.75 | 0.010435 |
| **70** | GPRIN3 | GPRIN family member 3 | 1.74 | 0.006705 |
| **71** | MAN1A1 | alpha-1,2-Mannosidase | 1.74 | 0.029347 |
| **72** | NDE1 | NudE neurodevelopment protein 1 | 1.74 | 0.048061 |
| **73** | RRH | Retinal pigment epithelium-derived rhodopsin homolog | 1.73 | 0.004696 |
| **74** | COL4A5 | Collagen type IV alpha 5 chain | 1.72 | 0.018037 |
| **75** | CAVIN2 | Caveolae associated protein 2 | 1.71 | 0.017033 |
| **76** | EIF5A2 | Eukaryotic translation initiation factor 5A | 1.68 | 0.031764 |
| **77** | EMILIN1 | Elastin microfibril interfacer 1 | 1.66 | 0.000281 |
| **78** | Cryba2 | BetaA2-crystallin | 1.64 | 0.046695 |
| **79** | SNX11 | Sorting nexin 11 | 1.63 | 0.005233 |
| **80** | DDX55 | ATP-dependent RNA helicase | 1.62 | 0.028715 |
| **81** | CTU1 | Cytoplasmic tRNA 2-thiolation protein 1 | 1.61 | 0.032253 |
| **82** | TPM2.1 | Tropomyosin 2 | 1.61 | 0.042138 |
| **83** | FAM126B | Family with sequence similarity 126 member B | 1.57 | 0.047324 |
| **84** | NUDT16L1 | Nudix hydrolase 16 like 1 | 1.54 | 0.006292 |
| **85** | CALCOCO1 | Calcium binding and coiled-coil domain 1 | 1.51 | 0.046253 |
| **86** | GUK1 | guanylate kinase | 1.51 | 0.006823 |
| **87** | NDUFAF3 | NADH dehydrogenase [ubiquinone] 1 alpha subcomplex assembly factor 3 | 1.50 | 0.014327 |
| **88** | EMILIN2 | Elastin microfibril interfacer 2 | 1.49 | 0.011787 |
| **89** | Cryba4 | BetaA4-crystallin | 1.48 | 0.022614 |
| **90** | DAPL1 | Death associated protein like 1 | 1.46 | 0.022380 |
| **91** | EIF4ENIF1 | Eukaryotic translation initiation factor 4E nuclear import factor 1 | 1.43 | 0.033000 |
| **92** | FILIP1L | Filamin A interacting protein 1 like | 1.40 | 0.017021 |
| **93** | LDB1 | LIM domain binding 1 | 1.40 | 0.024212 |
| **94** | DCAKD | Dephospho-CoA kinase domain containing | 1.39 | 0.005231 |
| **95** | PTGDS | Prostaglandin D2 synthase | 1.39 | 0.049460 |
| **96** | Crybb3 | BetaB3-crystallin | 1.36 | 0.040379 |
| **97** | TYR | Tyrosinase | 1.36 | 0.004466 |
| **98** | BORCS5 | BLOC-1-related complex subunit 5 | 1.35 | 0.014316 |
| **99** | BRD8 | Bromodomain containing 8 | 1.34 | 0.041150 |
| **100** | USF2 | Upstream transcription factor 2, c-fos interacting | 1.34 | 0.012286 |
| **101** | MYL9 | Myosin light chain 9 | 1.24 | 0.001223 |
| **102** | TCF12 | Transcription factor 12 | 1.20 | 0.019926 |
| **103** | ITPKB | Kinase | 1.16 | 0.042953 |
| **104** | SNX7 | Sorting nexin 7 | 1.15 | 0.029730 |
| **105** | CD248 | CD248 molecule | 1.13 | 0.021681 |
| **106** | RPS6KB2 | non-specific serine/threonine protein kinase | 1.12 | 0.027705 |
| **107** | CARHSP1 | Calcium regulated heat stable protein 1 | 1.10 | 0.047331 |
| **108** | CDKN1B | Cyclin dependent kinase inhibitor 1B | 1.08 | 0.012633 |
| **109** | PLBD1 | Phospholipase B-like | 1.07 | 0.017888 |
| **110** | S1PR2 | Sphingosine-1-phosphate receptor 2 | 1.04 | 0.017898 |
| **111** | MRPS5 | Mitochondrial ribosomal protein S5 | 1.03 | 0.048271 |
| **112** | AGT | Angiotensinogen | 1.02 | 0.003075 |
| **113** | DCT | Dopachrome tautomerase | 1.00 | 0.032798 |
| **114** | PARP14 | Poly [ADP-ribose] polymerase | −1.01 | 0.031301 |
| **115** | ANKRD28 | Ankyrin repeat domain 28 | −1.03 | 0.014547 |
| **116** | AEBP1 | AE binding protein 1 | −1.04 | 0.018730 |
| **117** | FBLN7 | Fibulin 7 | −1.04 | 0.027783 |
| **118** | NAA35 | N-alpha-acetyltransferase 35, NatC auxiliary subunit | −1.04 | 0.002466 |
| **119** | SAAL1 | Serum amyloid A like 1 | −1.05 | 0.022758 |
| **120** | PLA2G4A | Phospholipase A2 | −1.08 | 0.047823 |
| **121** | LAS1L | LAS1 like ribosome biogenesis factor | −1.09 | 0.004832 |
| **122** | RASSF8 | Ras association domain family member 8 | −1.09 | 0.048608 |
| **123** | SLC12A9 | Solute carrier family 12 member 9 | −1.12 | 0.045729 |
| **124** | TBC1D8B | TBC1 domain family member 8B | −1.12 | 0.018603 |
| **125** | IFT81 | Intraflagellar transport 81 | −1.16 | 0.023962 |
| **126** | PTPRC | protein-tyrosine-phosphatase | −1.17 | 0.004390 |
| **127** | NDUFB6 | NADH dehydrogenase [ubiquinone] 1 beta subcomplex subunit 6 | −1.18 | 0.004060 |
| **128** | FHOD3 | Formin homology 2 domain containing 3 | −1.21 | 0.029769 |
| **129** | DDX58 | RNA helicase | −1.22 | 0.031446 |
| **130** | LOC100735737 | Guanylate cyclase domain-containing protein | −1.22 | 0.035107 |
| **131** | ZMYM3 | Zinc finger MYM-type containing 3 | −1.22 | 0.005127 |
| **132** | RALGAPA2 | Ral GTPase activating protein catalytic alpha subunit 2 | −1.23 | 0.015144 |
| **133** | ATRN | Attractin | −1.27 | 0.044697 |
| **134** | MCM7 | DNA replication licensing factor MCM7 | −1.28 | 0.038024 |
| **135** | FMNL1.1 | Formin like 1 | −1.30 | 0.003436 |
| **136** | PECAM1 | Platelet and endothelial cell adhesion molecule 1 | −1.30 | 0.012055 |
| **137** | MBP | Myelin basic protein | −1.33 | 0.042358 |
| **138** | GGA3 | Golgi associated, gamma adaptin ear containing, ARF binding protein 3 | −1.34 | 0.042754 |
| **139** | ATP2A3 | Calcium-transporting ATPase | −1.38 | 0.028647 |
| **140** | DNM3 | dynamin GTPase | −1.38 | 0.030830 |
| **141** | LRCH4.1 | Leucine rich repeats and calponin homology domain containing 4 | −1.41 | 0.015327 |
| **142** | VAMP8 | Vesicle associated membrane protein 8 | −1.47 | 0.021599 |
| **143** | C2orf69 | Chromosome 2 open reading frame 69 | −1.48 | 0.037749 |
| **144** | KDM5A | [histone H3]-trimethyl-L-lysine(4) demethylase | −1.48 | 0.047823 |
| **145** | LIFR | LIF receptor subunit alpha | −1.50 | 0.046997 |
| **146** | KPNA2 | Importin subunit alpha | −1.51 | 0.013159 |
| **147** | DGKH | Diacylglycerol kinase | −1.53 | 0.004551 |
| **148** | DNAAF10 | WD repeat domain 92 | −1.54 | 0.021497 |
| **149** | ZNF280C | Zinc finger protein 280C | −1.54 | 0.002625 |
| **150** | LMOD1 | Leiomodin 1 | −1.56 | 0.024823 |
| **151** | ITGA9 | Integrin subunit alpha 9 | −1.57 | 0.000808 |
| **152** | PURG | Purine rich element binding protein G | −1.57 | 0.046618 |
| **153** | RCAN2 | Calcipressin-2 | −1.58 | 0.004371 |
| **154** | SLC39A6 | Solute carrier family 39 member 6 | −1.59 | 0.008337 |
| **155** | CNTNAP1 | Contactin associated protein 1 | −1.60 | 0.010512 |
| **156** | PLPPR4 | Phospholipid phosphatase related 4 | −1.64 | 0.012625 |
| **157** | FAS | Tumor necrosis factor receptor superfamily member 6 | −1.70 | 0.018933 |
| **158** | FOXO3 | Forkhead box O3 | −1.70 | 0.017970 |
| **159** | GAD2 | Glutamate decarboxylase 2 | −1.71 | 0.039751 |
| **160** | KDELR2 | ER lumen protein-retaining receptor | −1.74 | 0.032573 |
| **161** | PABIR1 | Family with sequence similarity 122A | −1.74 | 0.000195 |
| **162** | PMP2 | Myelin P2 protein | −1.76 | 0.019565 |
| **163** | ARNTL | Aryl hydrocarbon receptor nuclear translocator like | −1.78 | 0.035556 |
| **164** | FBXW9 | F-box and WD repeat domain containing 9 | −1.78 | 0.033769 |
| **165** | SAMD9 | Sterile alpha motif domain containing 9 | −1.80 | 0.041110 |
| **166** | NSRP1 | Nuclear speckle splicing regulatory protein 1 | −1.81 | 0.036335 |
| **167** | DOCK4 | Dedicator of cytokinesis 4 | −1.82 | 0.043766 |
| **168** | UBAC1 | Ubiquitin-associated domain-containing protein 1 | −1.84 | 0.032207 |
| **169** | CNTROB | Centrobin, centriole duplication and spindle assembly protein | −1.93 | 0.037068 |
| **170** | INTS8 | Integrator complex subunit 8 | −1.94 | 0.016624 |
| **171** | CD53 | Tetraspanin | −1.96 | 0.044523 |
| **172** | ZNF512B | Zinc finger protein 512B | −1.96 | 0.006011 |
| **173** | LMBRD1 | LMBR1 domain containing 1 | −2.01 | 0.013281 |
| **174** | CPNE6 | Copine 6 | −2.18 | 0.031013 |
| **175** | ITM2C | Integral membrane protein 2 | −2.19 | 0.002969 |
| **176** | HYPK | Huntingtin interacting protein K | −2.20 | 0.007233 |
| **177** | SRD5A3 | Polyprenol reductase | −2.21 | 0.025470 |
| **178** | ACD | ACD shelterin complex subunit and telomerase recruitment factor | −2.24 | 0.039790 |
| **179** | NPHP1 | Nephrocystin 1 | −2.31 | 0.001586 |
| **180** | SH3GL3 | SH3 domain containing GRB2 like 3, endophilin A3 | −2.31 | 0.017625 |
| **181** | DOCK8 | Dedicator of cytokinesis 8 | −2.42 | 0.002730 |
| **182** | STAB1 | Stabilin 1 | −2.44 | 0.001409 |
| **183** | FMN1 | Formin 1 | −2.46 | 0.028122 |
| **184** | NTM | Neurotrimin | −2.46 | 0.045223 |
| **185** | ARHGAP30 | Rho GTPase activating protein 30 | −2.50 | 0.009533 |
| **186** | ARL13B | ADP ribosylation factor like GTPase 13B | −2.60 | 0.032918 |
| **187** | IMMT | MICOS complex subunit MIC60 | −2.61 | 0.038593 |
| **188** | SLC37A4 | Solute carrier family 37 member 4 | −2.64 | 0.045793 |
| **189** | GFRA1 | GDNF family receptor alpha-1 | −2.65 | 0.002120 |
| **190** | ISG20L2 | Interferon stimulated exonuclease gene 20 like 2 | −2.69 | 0.014801 |
| **191** | MSANTD2 | Myb/SANT DNA binding domain containing 2 | −2.78 | 0.003985 |
| **192** | DDX49 | RNA helicase | −2.80 | 0.018214 |
| **193** | ZMIZ1 | Zinc finger MIZ-type containing 1 | −2.81 | 0.004385 |
| **194** | RUNDC3A | RUN domain containing 3A | −2.86 | 0.000464 |
| **195** | TOP2A | DNA topoisomerase 2 | −3.07 | 0.001099 |
| **196** | ADGRB3 | Adhesion G protein-coupled receptor B3 | −3.09 | 0.016227 |
| **197** | CDH23 | Cadherin related 23 | −3.10 | 0.002670 |
| **198** | DSG1 | Desmoglein 1 | −3.29 | 0.037365 |
| **199** | CHCHD10 | Coiled-coil-helix-coiled-coil-helix domain containing 10 | −3.43 | 0.027798 |
| **200** | LOC100723477 | Methyltransferase like 17 | −3.79 | 0.023788 |
| **201** | BCL7A | BAF chromatin remodeling complex subunit BCL7A | −3.81 | 0.022338 |
| **202** | VSIG10 | V-set and immunoglobulin domain containing 10 | −4.59 | 0.000911 |
| **203** | VKORC1 | vitamin-K-epoxide reductase (warfarin-sensitive) | −4.80 | 0.027468 |
|  |  |  |  |  |
| **C. NC** | | | | |
|  | **Gene name** | **Protein name** | **Log_2_ (FC)** | ***P* value** |
| **1** | BCL7A | BAF chromatin remodeling complex subunit BCL7A | 5.37 | 0.002849 |
| **2** | DNM1.1 | dynamin GTPase | 4.97 | 0.049655 |
| **3** | DMAC2L | Distal membrane arm assembly complex 2 like | 4.59 | 0.002313 |
| **4** | Rab11fip1 | RAB11 family interacting protein 1 | 4.14 | 0.023919 |
| **5** | IL4I1 | Amine oxidase | 3.97 | 0.031891 |
| **6** | CDH4 | Cadherin 4 | 3.82 | 0.000485 |
| **7** | RAI14 | Retinoic acid induced 14 | 3.68 | 0.000459 |
| **8** | GTF2A1 | General transcription factor IIA subunit 1 | 3.56 | 0.032442 |
| **9** | GSTCD | Glutathione S-transferase C-terminal domain-containing protein | 3.49 | 0.005417 |
| **10** | SYNGR2 | Synaptogyrin 2 | 3.40 | 0.014075 |
| **11** | RB1 | RB transcriptional corepressor 1 | 3.17 | 0.003809 |
| **12** | OTUB2 | ubiquitinyl hydrolase 1 | 3.12 | 0.040985 |
| **13** | ACD | ACD shelterin complex subunit and telomerase recruitment factor | 3.05 | 0.008272 |
| **14** | ZNF664 | Zinc finger protein 664 | 3.00 | 0.027573 |
| **15** | GSPT2 | G1 to S phase transition 2 | 2.98 | 0.028719 |
| **16** | DAB1 | DAB adaptor protein 1 | 2.91 | 0.029504 |
| **17** | SH2D3C | SH2 domain containing 3C | 2.82 | 0.030718 |
| **18** | CFAP36 | Cilia- and flagella-associated protein 36 | 2.76 | 0.023462 |
| **19** | GPN3 | GPN-loop GTPase 3 | 2.64 | 0.000068 |
| **20** | SV2C | Synaptic vesicle glycoprotein 2C | 2.64 | 0.027996 |
| **21** | TPRN | Taperin | 2.64 | 0.030314 |
| **22** | KCNAB1 | Voltage-gated potassium channel subunit beta-1 | 2.59 | 0.005389 |
| **23** | HYPK | Huntingtin interacting protein K | 2.54 | 0.002777 |
| **24** | CPD | Carboxypeptidase D | 2.54 | 0.042792 |
| **25** | POLR1A | DNA-directed RNA polymerase subunit | 2.53 | 0.031091 |
| **26** | GABRR1 | GABA(C) receptor | 2.49 | 0.033864 |
| **27** | ZFAND3 | Zinc finger AN1-type containing 3 | 2.47 | 0.011553 |
| **28** | SLC2A12 | Solute carrier family 2 member 12 | 2.46 | 0.021065 |
| **29** | SPRYD3 | SPRY domain containing 3 | 2.46 | 0.026748 |
| **30** | GNG3 | Guanine nucleotide-binding protein subunit gamma | 2.45 | 0.035399 |
| **31** | CNGB3 | Cyclic nucleotide gated channel subunit beta 3 | 2.44 | 0.000318 |
| **32** | GOLT1B | Golgi transport 1B | 2.36 | 0.026805 |
| **33** | TMA16 | Translation machinery-associated protein 16 | 2.34 | 0.026369 |
| **34** | ZFP37 | ZFP37 zinc finger protein | 2.30 | 0.006048 |
| **35** | DHPS | Deoxyhypusine synthase | 2.30 | 0.048917 |
| **36** | FAT1 | FAT atypical cadherin 1 | 2.29 | 0.015619 |
| **37** | ALG5 | dolichyl-phosphate beta-glucosyltransferase | 2.27 | 0.025152 |
| **38** | ADAP2 | ArfGAP with dual PH domains 2 | 2.26 | 0.047710 |
| **39** | WNK2 | non-specific serine/threonine protein kinase | 2.24 | 0.025687 |
| **40** | SGSM1 | Small G protein signaling modulator 1 | 2.16 | 0.012277 |
| **41** | MED14 | Mediator of RNA polymerase II transcription subunit 14 | 2.13 | 0.047720 |
| **42** | DMWD | DM1 locus, WD repeat containing | 2.06 | 0.021423 |
| **43** | Higd2a | HIG1 domain-containing protein | 2.05 | 0.045987 |
| **44** | NAT14 | N-acetyltransferase 14 (putative) | 2.05 | 0.044138 |
| **45** | UBAC1 | Ubiquitin-associated domain-containing protein 1 | 2.05 | 0.018943 |
| **46** | LINGO1 | Leucine rich repeat and Ig domain containing 1 | 2.04 | 0.005623 |
| **47** | RETREG2 | Reticulophagy regulator family member 2 | 2.02 | 0.006007 |
| **48** | PHLDA1 | Pleckstrin homology like domain family A member 1 | 2.02 | 0.024108 |
| **49** | KDM1B | Lysine demethylase 1B | 2.00 | 0.015573 |
| **50** | VPS53 | Vacuolar protein sorting-associated protein 53 homolog | 2.00 | 0.013221 |
| **51** | PDE8A | Phosphodiesterase | 1.99 | 0.000983 |
| **52** | PARVB | Parvin beta | 1.99 | 0.013323 |
| **53** | IGSF3 | Immunoglobulin superfamily member 3 | 1.98 | 0.011358 |
| **54** | ACKR3 | Atypical chemokine receptor 3 | 1.97 | 0.012970 |
| **55** | SV2B | Synaptic vesicle glycoprotein 2B | 1.96 | 0.028855 |
| **56** | PCF11 | PCF11 cleavage and polyadenylation factor subunit | 1.95 | 0.007063 |
| **57** | PHF14 | PHD finger protein 14 | 1.94 | 0.000853 |
| **58** | CDK2AP2 | Cyclin-dependent kinase 2-associated protein | 1.94 | 0.031983 |
| **59** | RBM33 | RNA binding motif protein 33 | 1.94 | 0.012793 |
| **60** | DMD.1 | Dystrophin | 1.91 | 0.040102 |
| **61** | MTIF2 | Mitochondrial translational initiation factor 2 | 1.91 | 0.034676 |
| **62** | NMNAT3 | Nicotinamide-nucleotide adenylyltransferase | 1.83 | 0.013285 |
| **63** | TIMM21 | Mitochondrial import inner membrane translocase subunit Tim21 | 1.83 | 0.032052 |
| **64** | RASGRF2 | Ras protein specific guanine nucleotide releasing factor 2 | 1.82 | 0.001048 |
| **65** | RANBP9 | RAN binding protein 9 | 1.78 | 0.044515 |
| **66** | STK32C | Serine/threonine kinase 32C | 1.74 | 0.043894 |
| **67** | SLC6A9 | Transporter | 1.73 | 0.024059 |
| **68** | TCF4 | Transcription factor 4 | 1.73 | 0.038001 |
| **69** | AP5S1 | Adaptor related protein complex 5 subunit sigma 1 | 1.71 | 0.041618 |
| **70** | HSD17B8 | Hydroxysteroid 17-beta dehydrogenase 8 | 1.69 | 0.006464 |
| **71** | CBX1 | Chromobox 1 | 1.68 | 0.031921 |
| **72** | LIMCH1 | LIM and calponin homology domains 1 | 1.67 | 0.039733 |
| **73** | BIRC2 | Baculoviral IAP repeat containing 2 | 1.62 | 0.045582 |
| **74** | MOV10 | RNA helicase | 1.61 | 0.016618 |
| **75** | Septin4.2 | Septin | 1.61 | 0.027705 |
| **76** | KRIT1 | KRIT1 ankyrin repeat containing | 1.59 | 0.035381 |
| **77** | IL33 | Interleukin-33 | 1.57 | 0.004797 |
| **78** | PLAT | Plasminogen activator | 1.56 | 0.017674 |
| **79** | NPHP1 | Nephrocystin 1 | 1.55 | 0.019349 |
| **80** | PDE6C | Phosphodiesterase | 1.55 | 0.015436 |
| **81** | CHD2 | DNA helicase | 1.54 | 0.006396 |
| **82** | ZNF84 | Zinc finger protein 84 | 1.53 | 0.010515 |
| **83** | IRAG1 | Murine retrovirus integration site 1 homolog | 1.51 | 0.019419 |
| **84** | Pigz | Mannosyltransferase | 1.48 | 0.032913 |
| **85** | SIK3 | SIK family kinase 3 | 1.46 | 0.022941 |
| **86** | TAS1R1 | Taste 1 receptor member 1 | 1.44 | 0.039531 |
| **87** | LMOD1 | Leiomodin 1 | 1.39 | 0.041175 |
| **88** | WWP2 | E3 ubiquitin-protein ligase | 1.39 | 0.019696 |
| **89** | Krt13 | Keratin 13 | 1.31 | 0.044365 |
| **90** | MYO5B | Myosin VB | 1.31 | 0.016698 |
| **91** | CNTNAP1 | Contactin associated protein 1 | 1.30 | 0.031218 |
| **92** | HARS2 | histidine--tRNA ligase | 1.30 | 0.040042 |
| **93** | VAV3 | Vav guanine nucleotide exchange factor 3 | 1.27 | 0.029163 |
| **94** | SHPRH | SNF2 histone linker PHD RING helicase | 1.21 | 0.031897 |
| **95** | MRPL16 | 39S ribosomal protein L16, mitochondrial | 1.17 | 0.029080 |
| **96** | RAB3IP | RAB3A interacting protein | 1.16 | 0.030025 |
| **97** | NEK1 | NIMA related kinase 1 | 1.15 | 0.030044 |
| **98** | DAB2IP | DAB2 interacting protein | 1.13 | 0.014692 |
| **99** | FGL2 | Fibrinogen like 2 | 1.13 | 0.009290 |
| **100** | CDK6 | Cyclin dependent kinase 6 | 1.12 | 0.046663 |
| **101** | DIMT1 | rRNA adenine N(6)-methyltransferase | 1.12 | 0.037133 |
| **102** | ADD2 | Adducin 2 | 1.08 | 0.042789 |
| **103** | SENP7 | SUMO specific peptidase 7 | 1.07 | 0.023705 |
| **104** | GDAP1 | Ganglioside induced differentiation associated protein 1 | 1.06 | 0.030088 |
| **105** | PRRC1 | Proline rich coiled-coil 1 | 1.06 | 0.015120 |
| **106** | PIK3CB | phosphatidylinositol-4,5-bisphosphate 3-kinase | 1.05 | 0.026607 |
| **107** | SFXN2 | Sidoreflexin | 1.04 | 0.020182 |
| **108** | LRRC8D | Leucine rich repeat containing 8 VRAC subunit D | 1.03 | 0.008858 |
| **109** | PRAF2 | PRA1 family protein | 1.00 | 0.021809 |
| **110** | CDIPT | CDP-diacylglycerol--inositol 3-phosphatidyltransferase | −1.00 | 0.007730 |
| **111** | DERL1 | Derlin | −1.02 | 0.001091 |
| **112** | SOS2 | SOS Ras/Rho guanine nucleotide exchange factor 2 | −1.03 | 0.035814 |
| **113** | LRG1 | Leucine rich alpha-2-glycoprotein 1 | −1.04 | 0.046761 |
| **114** | DOK2 | Docking protein 2 | −1.07 | 0.032732 |
| **115** | ZNF280C | Zinc finger protein 280C | −1.09 | 0.021293 |
| **116** | CASC3 | Protein CASC3 | −1.16 | 0.049587 |
| **117** | C8orf82 | Chromosome 8 open reading frame 82 | −1.17 | 0.047160 |
| **118** | Apf | Serpin domain-containing protein | −1.19 | 0.038652 |
| **119** | C5 | Complement C5 | −1.19 | 0.011393 |
| **120** | TTC5 | Tetratricopeptide repeat domain 5 | −1.20 | 0.029386 |
| **121** | ARHGAP23 | Rho GTPase activating protein 23 | −1.21 | 0.047451 |
| **122** | CDC123 | Cell division cycle protein 123 homolog | −1.22 | 0.002769 |
| **123** | TMED3 | Transmembrane p24 trafficking protein 3 | −1.24 | 0.011797 |
| **124** | MEST | Mesoderm specific transcript | −1.26 | 0.041182 |
| **125** | LRIT3 | Leucine rich repeat, Ig-like and transmembrane domains 3 | −1.27 | 0.046985 |
| **126** | FBLN7 | Fibulin 7 | −1.28 | 0.008938 |
| **127** | SPP2 | Secreted phosphoprotein 24 | −1.29 | 0.047260 |
| **128** | RABEPK | Rab9 effector protein with kelch motifs | −1.32 | 0.003774 |
| **129** | MATN2 | Matrilin 2 | −1.43 | 0.043164 |
| **130** | WDR46 | WD repeat domain 46 | −1.49 | 0.027608 |
| **131** | PARD6B | Par-6 family cell polarity regulator beta | −1.51 | 0.031382 |
| **132** | SLC39A6 | Solute carrier family 39 member 6 | −1.51 | 0.011165 |
| **133** | LOC100722818 | Cytochrome P450 | −1.52 | 0.043814 |
| **134** | FLAD1 | FAD synthase | −1.55 | 0.028507 |
| **135** | TSPAN5 | Tetraspanin | −1.56 | 0.032852 |
| **136** | GFRA2 | GDNF family receptor alpha-2 | −1.59 | 0.029516 |
| **137** | FAM118A | Family with sequence similarity 118 member A | −1.66 | 0.015512 |
| **138** | MAN1A1 | alpha-1,2-Mannosidase | −1.66 | 0.036338 |
| **139** | Krt12 | Keratin 12 | −1.68 | 0.013304 |
| **140** | ARNTL | Aryl hydrocarbon receptor nuclear translocator like | −1.69 | 0.045088 |
| **141** | MPST | Sulfurtransferase | −1.69 | 0.036233 |
| **142** | UMAD1 | UBAP1-MVB12-associated (UMA) domain containing 1 | −1.70 | 0.033075 |
| **143** | TRIP4 | Thyroid hormone receptor interactor 4 | −1.73 | 0.031539 |
| **144** | PAXIP1 | PAX interacting protein 1 | −1.74 | 0.044455 |
| **145** | RANBP6 | RAN binding protein 6 | −1.77 | 0.031008 |
| **146** | ATP1A4 | Sodium/potassium-transporting ATPase subunit alpha | −1.83 | 0.049418 |
| **147** | Col10a1 | C1q domain-containing protein | −1.86 | 0.024884 |
| **148** | SNAP47 | Synaptosomal-associated protein 47 | −1.87 | 0.000490 |
| **149** | CTNND2 | Catenin delta 2 | −1.87 | 0.036521 |
| **150** | WFIKKN2 | WAP, follistatin/kazal, immunoglobulin, kunitz and netrin domain containing 2 | −1.88 | 0.006941 |
| **151** | GPRIN1 | G protein regulated inducer of neurite outgrowth 1 | −1.89 | 0.043998 |
| **152** | SLC25A32 | Solute carrier family 25 member 32 | −1.91 | 0.046612 |
| **153** | TRIT1 | tRNA dimethylallyltransferase | −1.95 | 0.022172 |
| **154** | GCFC2 | GC-rich sequence DNA-binding factor 2 | −1.96 | 0.021542 |
| **155** | IFT80 | Intraflagellar transport 80 | −1.98 | 0.049955 |
| **156** | ASB3 | Ankyrin repeat and SOCS box containing 3 | −2.01 | 0.012102 |
| **157** | COL4A5 | Collagen type IV alpha 5 chain | −2.03 | 0.007142 |
| **158** | MYT1 | Myelin transcription factor 1 | −2.05 | 0.017165 |
| **159** | RBM48 | RNA-binding protein 48 | −2.07 | 0.025948 |
| **160** | SLC12A5 | Solute carrier family 12 member 5 | −2.08 | 0.045258 |
| **161** | TAF4 | TATA-box binding protein associated factor 4 | −2.12 | 0.038438 |
| **162** | TSR2 | Pre-rRNA-processing protein TSR2 homolog | −2.14 | 0.023263 |
| **163** | COL4A1 | Collagen type IV alpha 1 chain | −2.16 | 0.000058 |
| **164** | PROX1 | Prospero homeobox 1 | −2.16 | 0.043174 |
| **165** | HCN3 | Hyperpolarization activated cyclic nucleotide gated potassium channel 3 | −2.17 | 0.042439 |
| **166** | LTBP1 | Latent transforming growth factor beta binding protein 1 | −2.22 | 0.000337 |
| **167** | STX1A | Syntaxin 1A | −2.24 | 0.047304 |
| **168** | HMGA1 | High mobility group AT-hook 1 | −2.25 | 0.014057 |
| **169** | UXS1 | UDP-glucuronic acid decarboxylase 1 | −2.25 | 0.011266 |
| **170** | Dad1 | Dolichyl-diphosphooligosaccharide--protein glycosyltransferase subunit DAD1 | −2.27 | 0.022471 |
| **171** | DUSP19 | Dual specificity phosphatase 19 | −2.28 | 0.012793 |
| **172** | CDH23 | Cadherin related 23 | −2.32 | 0.016416 |
| **173** | CYB561D2 | ascorbate ferrireductase (transmembrane) | −2.35 | 0.012119 |
| **174** | THUMPD2 | THUMP domain containing 2 | −2.37 | 0.002130 |
| **175** | GDAP1L1 | Ganglioside induced differentiation associated protein 1 like 1 | −2.40 | 0.005141 |
| **176** | GRIFIN | Galectin | −2.41 | 0.035396 |
| **177** | SEZ6 | Seizure related 6 homolog | −2.44 | 0.002874 |
| **178** | ZSCAN2 | Zinc finger and SCAN domain containing 2 | −2.46 | 0.011579 |
| **179** | EMILIN2 | Elastin microfibril interfacer 2 | −2.49 | 0.000281 |
| **180** | COL8A2 | Collagen type VIII alpha 2 chain | −2.57 | 0.000143 |
| **181** | EXT2 | Exostosin glycosyltransferase 2 | −2.57 | 0.022938 |
| **182** | S100A14 | S100 calcium binding protein A14 | −2.58 | 0.005833 |
| **183** | PDE2A.1 | Phosphodiesterase | −2.60 | 0.010663 |
| **184** | COL8A1 | Collagen type VIII alpha 1 chain | −2.63 | 0.000088 |
| **185** | IDS | Iduronate 2-sulfatase | −2.63 | 0.027287 |
| **186** | PCNX1 | Pecanex-like protein | −2.67 | 0.014885 |
| **187** | CEP120 | Centrosomal protein 120 | −2.68 | 0.016032 |
| **188** | REEP1 | Receptor expression-enhancing protein | −2.68 | 0.035593 |
| **189** | PCDH10 | Protocadherin 10 | −2.75 | 0.026618 |
| **190** | EMILIN1 | Elastin microfibril interfacer 1 | −2.79 | 0.000001 |
| **191** | SNAP25.1 | Multifunctional fusion protein | −2.81 | 0.026158 |
| **192** | BFSP2 | Beaded filament structural protein 2 | −2.91 | 0.023959 |
| **193** | ACTBL2 | Actin beta like 2 | −3.09 | 0.023196 |
| **194** | ADAM15 | ADAM metallopeptidase domain 15 | −3.09 | 0.022754 |
| **195** | IFT27 | Intraflagellar transport 27 | −3.09 | 0.020748 |
| **196** | RORA | RAR related orphan receptor A | −3.10 | 0.006349 |
| **197** | MED11 | Mediator of RNA polymerase II transcription subunit 11 | −3.10 | 0.028208 |
| **198** | RRM2B | Ribonucleotide reductase regulatory TP53 inducible subunit M2B | −3.11 | 0.010327 |
| **199** | SUFU | Suppressor of fused homolog | −3.14 | 0.046885 |
| **200** | USF1 | Upstream transcription factor 1 | −3.22 | 0.023999 |
| **201** | EMILIN3 | Elastin microfibril interfacer 3 | −3.26 | 0.046201 |
| **202** | TENM2 | Teneurin transmembrane protein 2 | −3.29 | 0.006753 |
| **203** | COL4A2 | Collagen type IV alpha 2 chain | −3.38 | 0.000056 |
| **204** | BSCL2 | Seipin | −3.48 | 0.021273 |
| **205** | FBN1 | Fibrillin 1 | −3.49 | 0.000078 |
| **206** | ZMAT3 | Zinc finger matrin-type 3 | −3.51 | 0.025095 |
| **207** | DCX | Doublecortin | −3.57 | 0.004533 |
| **208** | ELMOD1 | ELMO domain containing 1 | −3.62 | 0.003453 |
| **209** | MFAP2 | Microfibril associated protein 2 | −3.62 | 0.000089 |
| **210** | GRIA3 | Glutamate receptor | −3.66 | 0.002048 |
| **211** | CDS1 | Phosphatidate cytidylyltransferase | −3.69 | 0.011421 |
| **212** | KLHL31 | Kelch like family member 31 | −3.88 | 0.010157 |
| **213** | NEXN | Nexilin F-actin binding protein | −4.23 | 0.023293 |
| **214** | GRM2 | Glutamate metabotropic receptor 2 | −4.48 | 0.025181 |
| **215** | SEC61G | Protein transport protein Sec61 subunit gamma | −4.51 | 0.015738 |
| **216** | ADAMTSL1 | ADAMTS like 1 | −4.54 | 0.000071 |
| **217** | SHISA9 | Shisa family member 9 | −4.68 | 0.000501 |
| **218** | CACNA1B | Voltage-dependent N-type calcium channel subunit alpha | −4.72 | 0.004639 |
| **219** | LOC100723477 | Methyltransferase like 17 | −4.81 | 0.006297 |
| **220** | PENK | Proenkephalin-A | −4.91 | 0.000221 |
| **221** | TSC22D1.1 | TSC22 domain family member 1 | −5.21 | 0.002322 |
| **222** | PLPPR3 | Phospholipid phosphatase related 3 | −5.54 | 0.000255 |
| **223** | COL4A4 | Collagen type IV alpha 4 chain | −6.51 | 0.000067 |
| **224** | B3GNT2 | Hexosyltransferase | −7.79 | 0.000003 |
| **225** | FBN2 | Fibrillin 2 | −8.26 | 0.000001 |

FC = fold change

| **Supplementary Table S2. List of differentially expressed proteins in common KEGG pathway.** | | | | | | |
| --- | --- | --- | --- | --- | --- | --- |
| **Pathway** | **NC** | | **ON** | | **OFF** | |
|  | **Gene name** | **Log_2_ (FC)** | **Gene name** | **Log_2_ (FC)** | **Gene name** | **Log_2_ (FC)** |
| **Protein digestion and absorption** | COL4A1 | −2.16 | COL4A1 | −1.50 | COL4A1 | 2.38 |
|  | COL4A2 | −3.38 | COL4A2 | −1.83 | COL4A2 | 2.52 |
|  | COL4A4 | −6.51 | COL4A4 | −4.64 | COL4A4 | 5.16 |
|  | COL8A1 | −2.63 | COL8A1 | −1.60 | COL8A1 | 2.27 |
|  | COL8A2 | −2.57 | COL8A2 | −2.07 | COL8A2 | 1.98 |
|  | COL4A5 | −2.03 | Col10a1 | −1.77 | COL4A5 | 1.72 |
|  | Col10a1 | −1.86 |  |  | SLC8A3 | 3.64 |
| **AGE-RAGE signaling pathway in diabetic complications** | COL4A1 | −2.16 | COL4A1 | −1.50 | COL4A1 | 2.38 |
|  | COL4A2 | −3.38 | COL4A2 | −1.83 | COL4A2 | 2.52 |
|  | COL4A4 | −6.51 | COL4A4 | −4.64 | COL4A4 | 5.16 |
|  | COL4A5 | −2.03 | NRAS | −3.90 | COL4A5 | 1.72 |
|  |  |  |  |  | AGT | 1.02 |
|  |  |  |  |  | CDKN1B | 1.08 |

FC = fold change; KEGG = Kyoto Encyclopedia of Genes and Genomes; NC = normal control
